# PD‐1 expressing islet‐specific CD4 ^+^ T cells promote bystander tolerance and prevent autoimmunity

**DOI:** 10.1111/imcb.70044

**Published:** 2025-07-07

**Authors:** Jeniffer D Loaiza Naranjo, Vivian Zhang, Rathna Ravichandran, Anne‐Sophie Bergot, Ranjeny Thomas, Emma E Hamilton‐Williams

**Affiliations:** ^1^ Frazer Institute The University of Queensland Brisbane QLD Australia

**Keywords:** autoimmunity, PD‐1, regulation, tolerance, type 1 diabetes

## Abstract

Loss of T‐cell tolerance to multiple islet antigens is a key feature of autoimmune type 1 diabetes. In this study, we investigated the requirement for programmed death 1 (PD‐1) expression by CD4^+^ T cells in the maintenance of self‐tolerance via bystander suppression of autoreactive CD8^+^ T cells using nonobese diabetic mice. We used CRISPR/Cas9 to selectively knockout PD‐1 in islet antigen‐specific BDC2.5 CD4^+^ T cells and observed the impact on bystander tolerance of 8.3 CD8^+^ T cells, specific for a different islet antigen. Loss of PD‐1 promoted the proliferation, Th1‐like effector‐memory phenotype, islet infiltration and expression of cytotoxic markers by BDC2.5 cells. PD‐1‐deficient BDC2.5 cells were impaired in their regulation of 8.3 cells, which proliferated more, developed an effector‐memory phenotype and increased expression of effector molecules. While antigen‐presenting cell maturation and migration into the pancreatic lymph node were not impacted by loss of PD‐1 expression from BDC2.5 cells, migration of BDC2.5 cells out of the lymph node was required for enhanced activation of the CD8^+^ T cells. Together, these events led to accelerated diabetes progression, suggesting that PD‐1 expression by CD4^+^ T cells promotes a tolerogenic microenvironment and restraining autoreactive CD8^+^ T cells.

## Graphical abstract

We investigated the requirement for programmed death 1 (PD‐1) expression by CD4^+^ T cells in the maintenance of self‐tolerance via bystander suppression of autoreactive CD8^+^ T cells. Using CRISPR/Cas9 to selectively knockout PD‐1 in islet antigen‐specific BDC2.5 CD4^+^ T cells, we demonstrate that loss of PD‐1 promoted the proliferation, Th1‐like effector‐memory phenotype, islet infiltration and expression of cytotoxic markers by BDC2.5 cells. PD‐1 deficient BDC2.5 cells were impaired in their regulation islet‐specific CD8^+^ T cells and accelerated autoimmune diabetes.

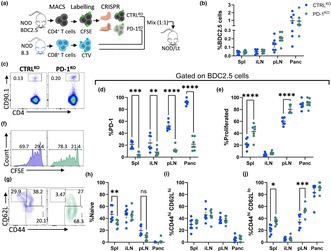

## INTRODUCTION

Unregulated activation of self‐reactive T cells targeting multiple islet antigens underlies autoimmune diseases such as type 1 diabetes (T1D). T1D is thought to be initiated by a loss of T‐cell tolerance toward insulin, but then spreads to target other islet‐beta cell‐derived proteins such as glutamic acid decarboxylase 65, chromogranin A (ChgA), islet‐specific glucose‐6‐phosphatase catalytic subunit‐related protein (IGRP) and others including neoantigens.^1^ A failure of multiple regulatory mechanisms leads to unrestrained autoreactive CD8^+^ T‐cell killing of the insulin‐producing beta cells.^2^ The molecular pathways through which islet antigen‐specific CD4^+^ T‐cell populations suppress CD8^+^ T cells specific for other islet antigens to prevent autoimmunity are not fully understood. Knowledge of these pathways will aid in the development of immunotherapies that restore immune regulation.

Programmed cell death 1 (PD‐1) is widely recognized as a major checkpoint inhibitor of T‐cell activation and effector function and is expressed by both CD4^+^ and CD8^+^ T cells. PD‐1 and programmed death ligand 1 (PD‐L1) blockade rapidly precipitates diabetes development in the nonobese diabetic (NOD) mouse model of T1D and reversed diabetes protection in immunotherapy‐treated NOD mice.^3^, ^4^ Recent work from our laboratory using antigen‐specific immunotherapy in NOD mice showed significant protection of diabetes progression by CD4^+^ type‐1 regulatory (Tr1) populations with sustained upregulation of PD‐1, accompanied by bystander tolerance of islet‐specific glucose‐6‐phosphatase catalytic subunit‐related protein (IGRP)‐specific CD8^+^ T cells.^5^ Induction of bystander tolerance, where T cells tolerized to one autoantigen can induce tolerance to other T‐cell reactivities, is a promising approach to tolerize multiple autoreactive T‐cell specificities simultaneously. Whether PD‐1 expression by CD4^+^ T cells is necessary for induction and maintenance of bystander tolerance is unknown.

Much of PD‐1 research has focused on cancer or infection models to reactivate PD‐1^+^ CD8^+^ exhausted T cells. While the role of PD‐1 in metabolism, T follicular helper (Tfh) cells and tolerance has been widely studied,^6^, ^7^, ^8^ less is known about PD‐1's function in regulatory CD4^+^ T cells, especially in Tr1 cells. A role for PD‐1 in CD4^+^ T cells during bystander tolerance has not yet been established. Notably, most *in vivo* studies investigating PD‐1 have used PD‐1 knockout (KO) mice or monoclonal antibody (mAb) to block PD‐1 or its ligands, both of which block PD‐1 interactions systemically.^9^ In these studies, it is not always clear which cell types are responsible for the PD‐1‐driven effects due to its expression on a diverse range of T‐cell subsets and activation states.^10^

Here, we designed a system to selectively knock out PD‐1, using clustered regularly interspaced short palindromic repeats (CRISPR)‐associated protein 9 (Cas9) technology, in BDC2.5 CD4^+^ T cells, specific for both an islet‐derived peptide from chromogranin A (ChgA_359‐372_) and a hybrid insulin peptide.^11^ We then determined the impact of loss of PD‐1 expression on the regulation of IGRP_206‐214_ peptide‐specific 8.3 CD8^+^ T cells.^12^, ^13^, ^14^, ^15^ We show that loss of PD‐1 on BDC2.5 cells enhanced the activation and effector function and migration into the pancreas of the BDC2.5 cells. This impaired their regulation of IGRP‐specific 8.3 T cells, which proliferated more and had increased effector function, resulting in accelerated diabetes progression. Thus, PD‐1 expression by antigen‐specific CD4^+^ T‐cell populations is required for bystander suppression of autoreactive CD8^+^ T cells of other specificities.

## RESULTS

### CRISPR‐targeted BDC2.5 cells acquire an activated memory phenotype in the absence of PD‐1

To investigate whether not if PD‐1 expression in BDC2.5 CD4^+^ T cells is required to maintain tolerance mechanisms *in vivo*, we used a recently developed approach to knock out PD‐1 on ex vivo isolated naïve T cells, via electroporation of sgRNA‐CRISPR/Cas9.^16^ This protocol allows for the study of tolerance, as the manipulated T cells remain in a naïve state before adoptive transfer without the need for *in vitro* culture.^16^ We knocked out (KO) PD‐1 in BDC2.5 CD4^+^ T cells or used a nontargeted control (CTRL^KO^) guide RNA, then tested the KO efficiency in BDC2.5 cells that were cultured with αCD3/CD28 beads for three days (Supplementary figure 1a). The loss of cell surface PD‐1 on BDC2.5 cells was highly efficient (Control 47–38% PD‐1^+^; PD‐1^KO^ 0–6% PD‐1^+^) (Supplementary figure 1b). Although incomplete inactivation of the PD‐1 gene cannot be discounted, the system was sufficiently efficient for further investigation of downstream tolerance outcomes.

**Figure 1:**
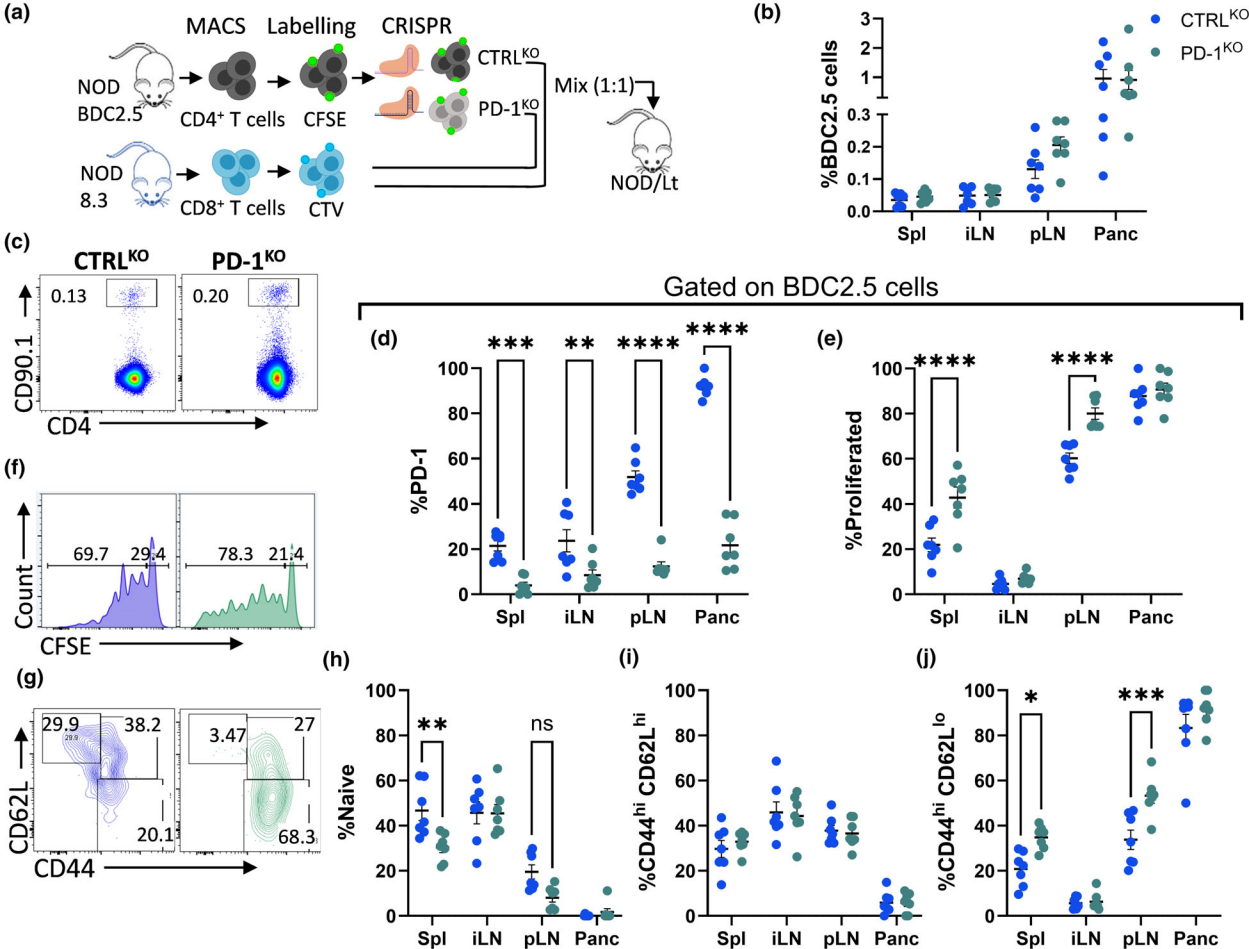
Enhanced proliferation and effector differentiation of BDC2.5 cells in the absence of PD‐1. **(a)** Experimental design. Purified CFSE‐labeled BDC2.5 cells were electroporated with untargeted CTRL (blue) or PD‐1 (green) sgRNA/Cas9 and coinjected with purified CTV‐labeled 8.3 cells into NOD mice. Spleen, iLN, pLN and pancreas were collected and analyzed four days after transfer. **(b)** Proportions of BDC2.5 cells with **(c)** representative dot plots. **(d)** Proportion of PD‐1 on BDC2.5 cells. **(e)** Proportion of divided BDC2.5 cells and **(f)** representative histograms. **(g)** Representative contour plots and frequencies of **(h)** naïve (CD62L^hi^ CD44^−^), **(i)** (CD62L^hi^ CD44^hi^) and **(j)** (CD62L^lo^ CD44^hi^) of BDC2.5 cells. Data are pooled from two experiments (*n* = 7 per group, mean ± SEM) using two‐way ANOVA with Sidak's multiple comparisons test. Flow cytometry plots examples are from pLN.

Next, we designed a cotransfer experiment to investigate the effect of PD‐1 deficiency in CD4^+^ T cells and their impact on bystander tolerance of CD8^+^ T cells. Purified CFSE‐labeled CTRL^KO^ or PD‐1^KO^ BDC2.5 cells were adoptively transferred together with CTV‐labeled 8.3 CD8^+^ T cells into NOD mice at 1:1 ratio (Figure 1a). Recipient mice were analyzed four days post‐transfer to allow for encounter with endogenous antigen in the pLN and migration of activated cells to the pancreas. There was no change in the proportion or total number of BDC2.5 cells in any tissues (Figure 1b, c; Supplementary figure 2a), suggesting the electroporation procedure did not impair cell survival or viability. BDC2.5/CTRL^KO^ cells upregulated PD‐1 in the pLN and pancreas in line with their activation by cognate islet antigen,^4^ while BDC2.5/PD‐1^KO^ cells had significantly lower PD‐1 expression (Figure 1d). Proliferating BDC2.5/PD‐1^KO^ cells were increased in both the spleen and pLNs (Figure 1e). In contrast, in the pancreas BDC2.5 cells had undergone extensive proliferation regardless of PD‐1 expression, confirming infiltration of BDC2.5 cells to the inflammation site is not restricted by PD‐1 expression (Figure 1e).^7^ However, BDC2.5/PD‐1^KO^ cells in all tissues including the pancreas had a higher proportion of cells that had undergone a high number of cell divisions (Figure 1f; Supplementary figure 2b, c). Loss of PD‐1 was evident in divided cells, specifically after 3–4 cell divisions (Supplementary figure 2d, e). In contrast to BDC2.5/CTRL^KO^ cells, naïve BDC2.5 PD‐1^KO^ cells rapidly differentiated into activated (CD44^hi^ CD62L^−^) cells in the spleen and pLN of recipient mice (Figure 1g–j). Activated and proliferated BDC2.5 cells did not accumulate as numbers did not change in the absence of PD‐1. Overall, these data suggest that lack of PD‐1 promotes proliferation and acquisition of an effector phenotype in islet antigen‐specific BDC2.5 cells.

### BDC2.5 cells develop an enhanced inflammatory phenotype in the absence of PD‐1

**Figure 2:**
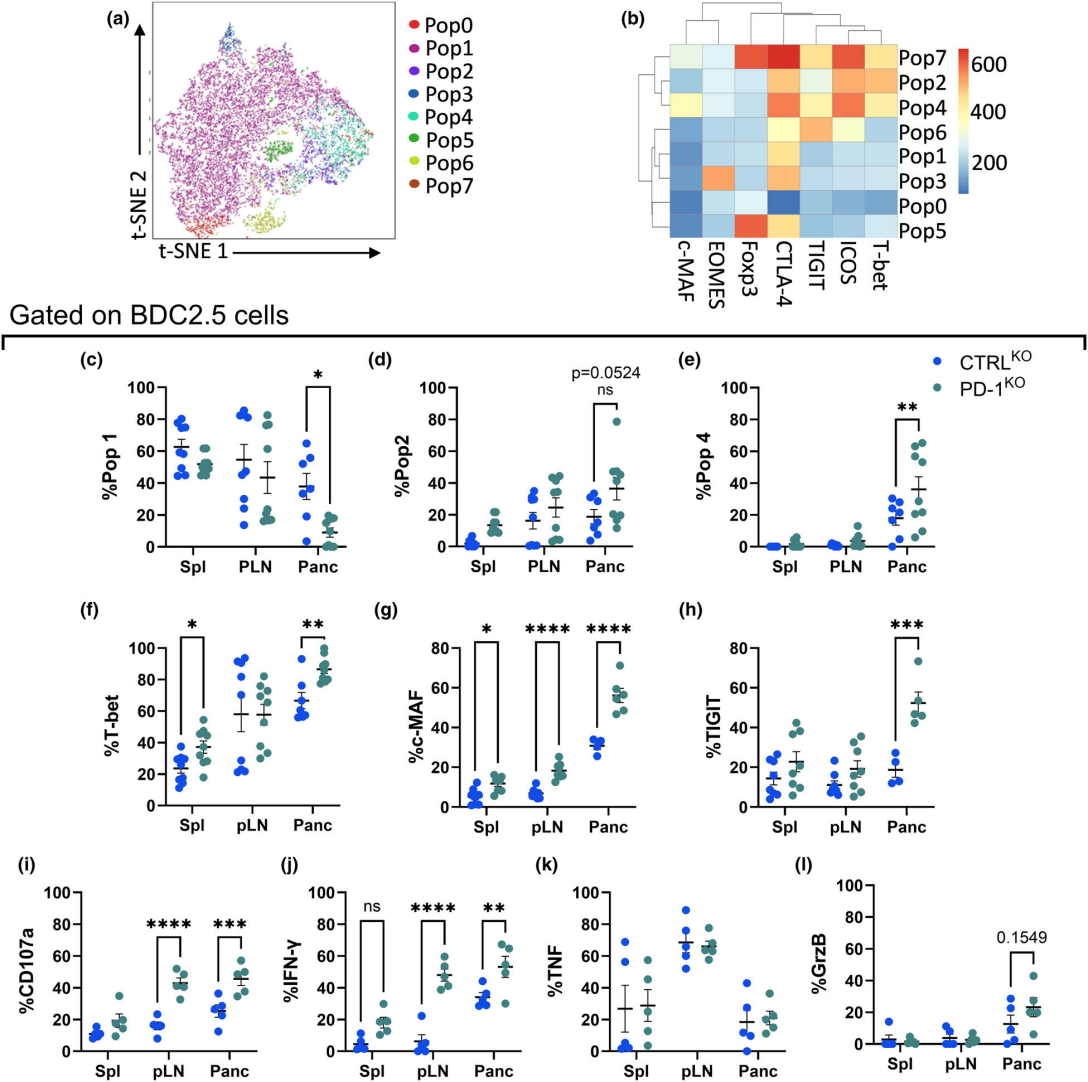
T helper 1 differentiation of BDC2.5 cells in the absence of PD‐1. Purified BDC2.5 cells were electroporated with CTRL (blue) or PD‐1 (green) sgRNA/Cas9 and cotransferred with purified 8.3 cells into NOD mice. Spleen, pLN and pancreas were analyzed on day 4. **(a)** t‐SNE and **(b)** heatmap of the populations identified in BDC2.5 cells by FlowSOM. Frequencies of **(c)** pop1, **(d)** pop2, **(e)** pop4 BDC2.5 cells. Proportions of **(f)** T‐bet, **(g)** c‐MAF and **(h)** TIGIT in BDC2.5 cells. Ten days post‐cotransfer, cells were collected and *ex vivo* stimulated with PMA/ionomycin. Frequencies of **(i)** CD107a, **(j)** IFN‐γ and **(k)** TNF in BDC2.5 cells. Data are pooled **(a–h)** or representative **(i–k)** of two experiments (*n* = 7 per group, mean ± SEM) using two‐way ANOVA with Sidak's multiple comparisons test.

To understand the impact of PD‐1 on the differentiation state of BDC2.5 cells, we characterized the BDC2.5 cells using a panel of transcription factors and immune‐modulatory markers four days post‐transfer. Using FlowSOM clustering, we identified eight populations (Figure 2a, b). Pop1, the largest population, was significantly higher among CTRL^KO^ BDC2.5 cells in the pancreas and was characterized by intermediate expression of intracellular CTLA‐4 and absence of expression of the other activation markers, suggesting an inactive status (Figure 2b; Supplementary figure 2e, f). PD‐1^KO^ BDC2.5 cells had a slight increase in pop2 (CTLA‐4^+^ T‐bet^+^ ICOS^+^) in the pancreas (36%) and pop4 (T‐bet^+^ ICOS^hi^ TIGIT^+^ CTLA‐4^hi^ c‐MAF^+^, 36%) (Figure 2b–e). Using traditional gating, we confirmed that BDC2.5/PD‐1^KO^ cells increased their expression of T‐bet and c‐MAF (Figure 2f–h), while there was no change in Foxp3 expression (Supplementary figure 2g, h). To confirm the differentiation of BDC2.5/PD‐1^KO^ cells into inflammatory Th lineages, we investigated their cytokine production and cytotoxic marker expression ten days post‐transfer after *ex vivo* stimulation. There was a significant increase in the number of BDC2.5/PD‐1^KO^ cells within the pancreas, with a similar trend in endogenous CD4^+^ T‐cell infiltration (Supplementary figure 3a, b). Ten days post‐transfer, BDC2.5/PD‐1^KO^ cells maintained their loss of PD‐1 expression (Supplementary figure 3c). Significantly higher proportions of CD107a in PD‐1^KO^ cells were observed in the pLN and pancreas and higher IFN‐γ overall, compared to their CTRL^KO^ counterparts (Figure 2i, j). CD107a is a lysosomal protein used to measure degranulation and cytotoxic activity in T cells.^17^ No changes were seen in inflammatory markers including TNF, granzyme B or IL‐17 in the PD‐1^KO^ cells (Figure 2k, l; Supplementary figure 3d–f). However, loss of PD‐1 resulted in a reduction in IL‐10 expression in the spleen four days post‐transfer (Supplementary figure 3e, f). Interestingly, endogenous CD4^+^ T cells displayed increased intracellular expression of granzyme B, but not other effector markers only in the pancreas (Supplementary figure 3g–j), suggesting a generalized increase in effector potential in CD4^+^ T cells that infiltrate the pancreas. Together these data suggest a role for PD‐1 in preventing inflammatory and cytotoxic self‐reactive CD4^+^ Th1 cell development.

### Lack of PD‐1 in BDC2.5 cells enhances proliferation and effector phenotype of 8.3 cells

**Figure 3:**
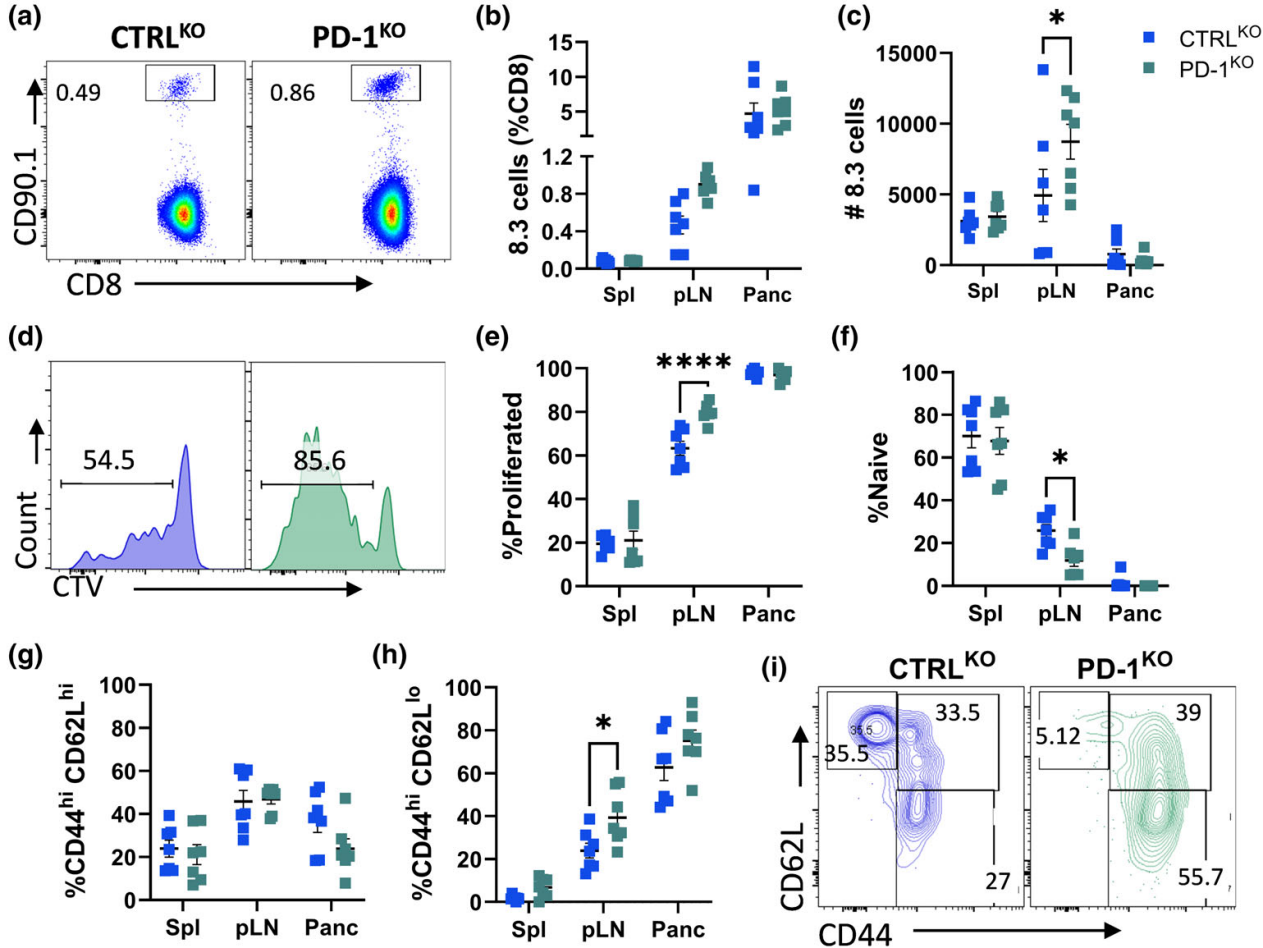
Enhanced proliferation and effector differentiation of IGRP‐specific 8.3 cells in the presence of BDC2.5 PD‐1^KO^ cells. CFSE‐labeled BDC2.5 cells were electroporated with CTRL (blue) or PD‐1 (green) sgRNA/Cas9 and coinjected with CTV‐labeled 8.3 cells into NOD mice. Spleen, pLN and pancreas were analyzed four days after transfer. **(a)** Representative dot plots, **(b)** proportions and **(c)** numbers of 8.3 cells. **(d)** Representative histograms and **(e)** proportions of proliferated 8.3 cells. Frequencies of **(f)** naïve (CD62L^hi^ CD44^−^), **(g)** (CD62L^hi^ CD44^hi^) and **(h)** (CD62L^lo^ CD44^hi^) and **(i)** representative contour plots of 8.3 cell populations. Data are pooled from two experiments (*n* = 7 per group, mean ± SEM) using two‐way ANOVA with Sidak's multiple comparisons test. Example FACS plots are from pLN.

In preclinical antigen‐specific immunotherapy models, Foxp3^−^ CD4^+^ regulatory (Tr1) T cells have been shown to suppress CD8^+^ T cells during the development of autoimmunity.^5^, ^18^ While BLIMP1 regulates the transition of Tfh cells to Tr1 and IL‐10 is required for disease suppression in some settings,^19^ the role of PD1 in bystander regulation of CD8^+^ T cells is unclear. To investigate this, we analyzed the 8.3 CD8^+^ T cells that were cotransferred with the CTRL or PD‐1^KO^ BDC2.5 cells. Four days after transfer, there were no differences in the proportion of 8.3 cells in any tissues, but the total number of 8.3 cells increased in pLN in the presence of PD‐1^KO^ BDC2.5 cells (Figure 3a–c). In line with this, the 8.3 cells proliferated at higher frequencies (Figure 3d, e) and differentiated into CD44^+^CD62L^−^ effector cells in the pLN when PD‐1^KO^ BDC2.5 cells were cotransferred (Figure 3f, h‐j). Thus, deficiency of PD‐1 in transferred BDC2.5 CD4^+^ T cells increased the proliferation and differentiation of effector CD8^+^ T cells in the pLN of recipient mice.

**Figure 4:**
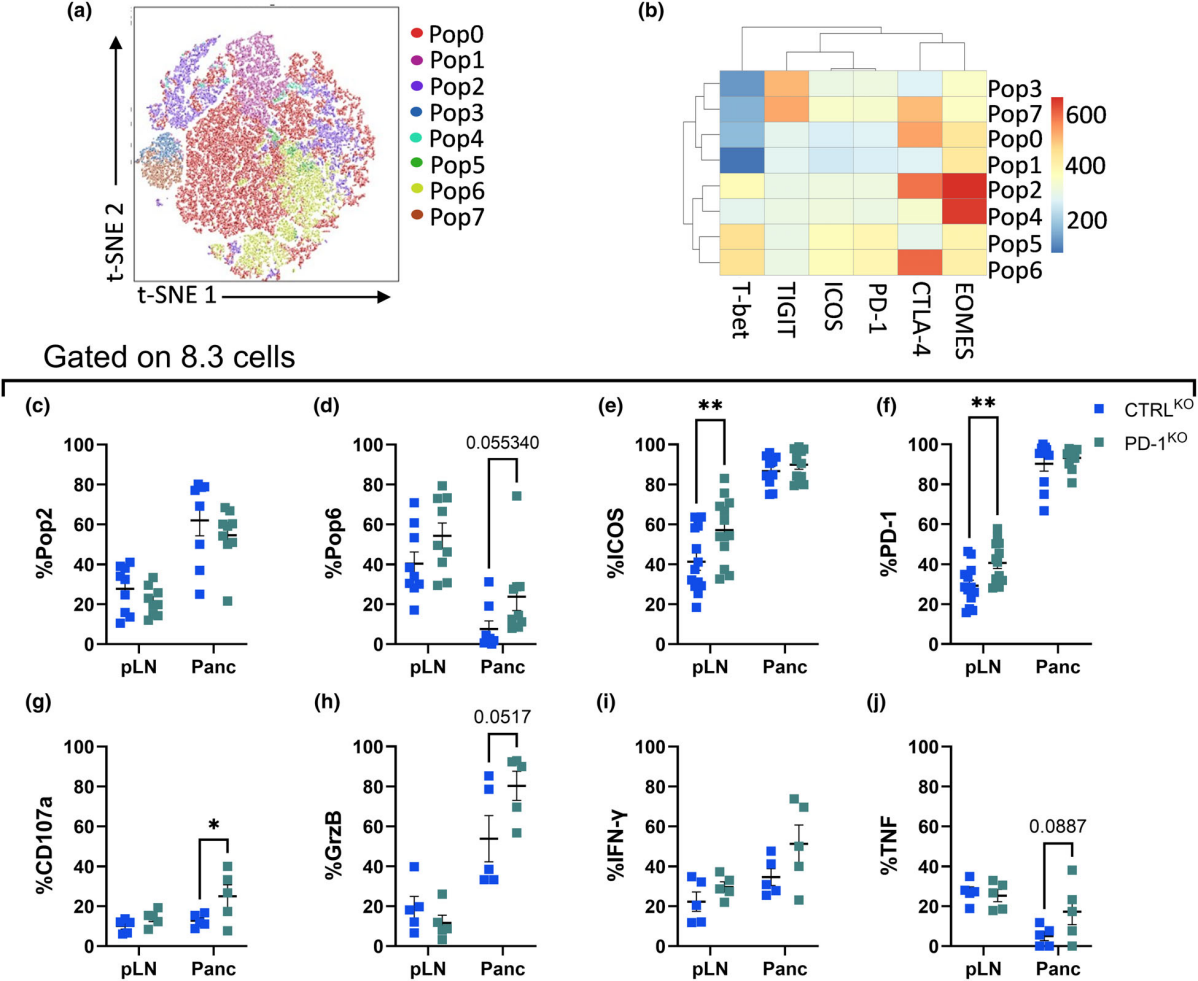
IGRP‐specific 8.3 cells increase the expression of effector molecules in the presence of BDC2.5 PD‐1^KO^ cells. Purified BDC2.5 cells electroporated with CTRL^KO^ (blue) or PD‐1^KO^ (green) were cotransferred with purified 8.3 cells into NOD mice. pLN and pancreas were analyzed 4 days post‐transfer. **(a)** t‐SNE and **(b)** heatmap of the populations identified in 8.3 cells by FlowSOM. Frequencies of 8.3 cells identified in **(c)** pop2 and **(d)** pop6. Frequencies of **(e)** ICOS and **(f)** PD‐1 in 8.3 cells. Ten days post‐cotransfer, cells were collected and *ex vivo* stimulated with PMA/ionomycin. Frequencies of **(g)** CD107a, **(h)** GrzB, **(i)** IFN‐γ and **(j)** TNF in 8.3 cells. Data are representative (a, b, g‐j) or pooled **(c–f)** from two experiments **(g–j)** (*n* = 5 or 9 per group, mean ± SEM) using the Mann–Whitney *t*‐test or two‐way ANOVA with Sidak's multiple comparisons test.

We further characterized 8.3 CD8^+^ T cells cotransferred with CTRL or BDC2.5/PD‐1^KO^ cells (Figure 4a, b). Of eight 8.3 CD8^+^ T‐cell populations identified with unsupervised FlowSOM, pop6 (T‐bet^+^ CTLA‐4^hi^ EOMES^lo^ PD‐1^+^ ICOS^+^) slightly increased in the pancreas of mice that received BDC2.5/PD‐1^KO^ cells relative to CTRL (Figure 4c, d; Supplementary figure 4a, b). Traditional gating confirmed the higher expression of ICOS and PD‐1 in the pLN of mice receiving BDC2.5/PD‐1^KO^ cells (Figure 4e, f), supporting increased antigen experience at this site.^20^, ^21^ We used the same adoptive transfer models to investigate cytokine responses at day ten, following *ex vivo* stimulation of lymphocytes with PMA/ionomycin for 4 h. The 8.3 cells located in the pancreas of mice that received BDC2.5/PD‐1^KO^ cells showed significant upregulation of CD107a and similar trends for Granzyme B and TNF (Figure 4g–j). A break in bystander tolerance with BDC2.5/PD‐1^KO^ cells was also evident in the endogenous CD8^+^ T cells, which increased granzyme B expression in the pancreas (Supplementary figure 4c–f). Thus, in mice receiving BDC2.5 CD4^+^ T cells lacking PD‐1, the activation and effector phenotype of both BDC2.5 and 8.3 cells was increased in the pLN.

### Lack of PD‐1 on BDC2.5 cells does not lead to antigen‐presenting cell maturation

**Figure 5:**
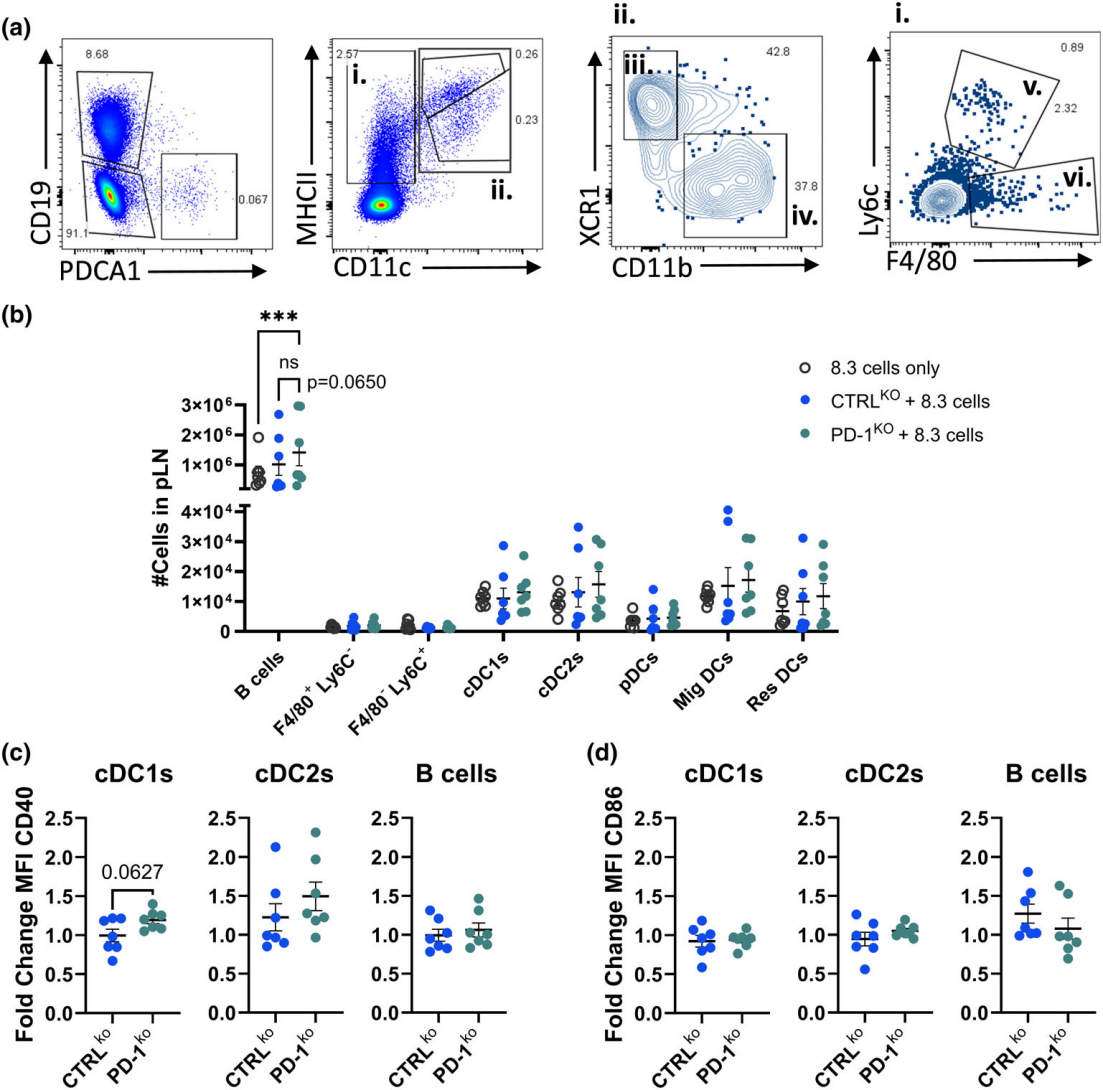
Increased number of B cells in the pLN in the presence of BDC2.5 PD‐1^KO^ cells. NOD mice received purified 8.3 cells alone or together with purified BDC2.5/CTRL^KO^ (blue) or PD‐1^KO^ (green) cells. pLNs were collected and analyzed four days after transfer. **(a)** Gating strategy of antigen‐presenting cells. CD3^−^CD56^−^ were gated on CD19^+^ PDCA1^−^ (B cells), PDCA‐1^+^ CD19^−^ (pDCs). CD19^−^ PDCA1^−^ were further gated on MHCII^hi^ CD11c^+^ (Migratory DCs); MHCII^int^ CD11c^+^ (resident DCs), MHCII^+^ CD11c^+^ XCR1^+^ CD11b^−^ (cDC1s); MHCII^+^ CD11c^+^ XCR1^−^ CD11b^+^ (cDC2s); MHCII^+^ CD11c^−^ F4/80^−^ Ly6c^+^; or MHCII^+^ CD11c^+^ F4/80^+^ Ly6c^−^. Fold change frequencies of maturation markers on **(b)** Absolute counts of B cells, F4/80^+^ Ly6c^−^, F4/80^lo^ Ly6c^+^, cDC1s, cDC2s, pDCs, migratory DCs and resident DCs. Fold change MFI of **(c)** CD40 and **(d)** CD86 in cDC1s, cDC2s and B cells. Data were normalized to the 8.3 cell‐only group. Data are pooled from two experiments (*n* = 6 or 7, mean ± SEM). **P‐value < 0.01 using two‐way ANOVA or Student's *t*‐test.

We next investigated whether the enhanced proliferation and activation of IGRP‐specific cells in the presence of BDC2.5/PD‐1^KO^ cells was due to increased activation signals from antigen‐presenting cells (APCs) cross‐activated by cognate CD4^+^ T cells. To test this, we used high dimensional flow cytometry to examine multiple APC populations and their maturation state,^22^ in the presence of BDC2.5 PD‐1^KO^ cells and 8.3^+^ CD8 T cells using the experimental design as Figure 1a. To control for the presence of CD8^+^ T cells in the system, we included a group of mice that received only 8.3 cells. T1D pathogenesis has been associated with elevated frequencies of inflammatory monocytes, macrophages and dendritic cells (DCs) that secrete pro‐inflammatory cytokines.^23^, ^24^, ^25^ TCRβ^−^ MHCII^+^ cells were gated to identify B cells (CD19^+^), plasmacytoid DCs (pDCs) (PDCA1^+^), CD19^−^ PDCA1‐ conventional (c)DC1s (CD11c^+^ XCR1^+^ SIRPα^−^), cDC2s (CD11c^+^ XCR1^−^ SIRPα^+^), Ly6c^+^ monocytes (CD11c^−^ Ly6c^+^ F480^−^), Ly6c^−^ F480^+^ cells, migratory DCs (MHCII^hi^ CD11c^lo^) and resident DCs (MHCII^lo^ CD11c^hi^) (Figure 5a). The number of B cells was significantly higher in the pLN of the BDC/PD‐1^KO^ group compared to the 8.3 cell‐only group, but was similar to the BDC/CTRL^KO^ group (Figure 5b). No other changes in the frequencies of APC populations were observed, including migratory and resident DC populations. No differences in the expression of activation markers CD40, CD86 (Figure 5c, d), or PD‐L1 and CD155 (Supplementary figure 5) were observed across any APC populations, including cDC1s that are known to specialize in cross‐presentation of tissue‐derived antigens to CD8^+^ T cells.^26^, ^27^ Thus, the increased proliferation and activation of the 8.3 cells in the pLN was not due to an increase in APC maturation status.

### Proliferative boost of 8.3 cells is prevented when BDC2.5 cells cannot access the pancreas

**Figure 6:**
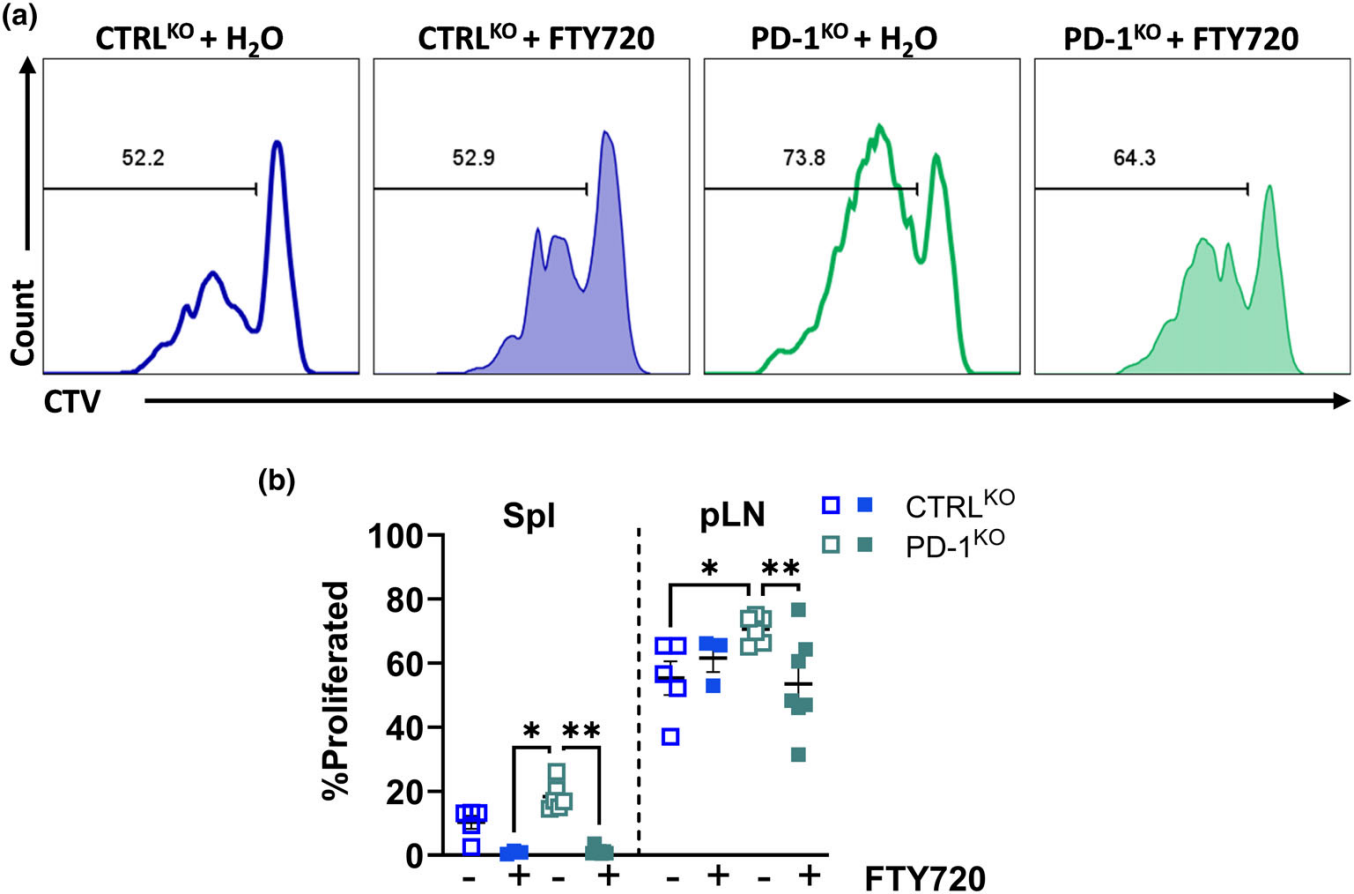
The proliferative boost of 8.3 cells requires migration of BDC2.5/PD‐1^KO^ cells. NOD mice received CFSE‐labeled BDC2.5 CTRL^KO^ (blue) or PD‐1^KO^ (green) cells together with CTV‐labeled 8.3 cells. Mice were treated with FTY720 or water at day‐1 and day 1 (i.p), spleen and pLN were collected at day 4. Representative histograms from pLN **(a)** and proportions **(b)** of proliferated 8.3 cells after FTY720 treatment. Data are representative from two experiments (*n* = 3 or 7 per group, mean ± SEM) using two‐way ANOVA with Tukey's correction.

As we did not find significant changes on the APC compartment, we hypothesized that the indirect activation of 8.3 cells seen in the pLN was a result of increased cytotoxicity and pro‐inflammatory activity of BDC2.5/PD‐1^KO^ cells in the pancreas, which would increase beta cell antigen release to the draining pLN, thereby increasing 8.3 cell proliferation. To test this, we treated NOD mice with FTY720, an inhibitor of lymphocyte‐specific egress from secondary lymphoid organs,^28^ or water one day before and one day post‐cotransfer of CTV‐labeled 8.3 cells and CFSE‐labeled BDC2.5/CTRL^KO^ or BDC2.5/PD‐1^KO^. Four days later, we saw that total splenic cells and total T cells from FTY720‐treated mice had lower viability than water‐treated mice (Supplementary figure 6c), a previously reported pro‐apoptotic effect of FTY720 on T cells.^29^ The opposite trend was seen on transferred cells, where accumulation in the spleen confirmed that FTY720 treatment prevents lymphocyte egress from lymphoid tissue (Supplementary figure 6d, e). Very low numbers of the transferred cells reached the pancreas in the FTY720‐treated mice, possibly due to the presence of tertiary lymphoid structures in the pancreas^30^ allowing entry of a small number of the transferred cells. Importantly, we found that FTY720 treatment prevented the proliferative boost of 8.3 cells in the presence of BDC2.5/PD‐1KO cells (Figure 6). This suggests that BDC2.5/PD‐1^KO^ cells need to migrate to the pancreas to drive inflammation and enhance the activation of the 8.3 cells.

### Loss of PD‐1 from BDC2.5 CD4 ^+^ T cells accelerates diabetes in NOD mice

**Figure 7:**
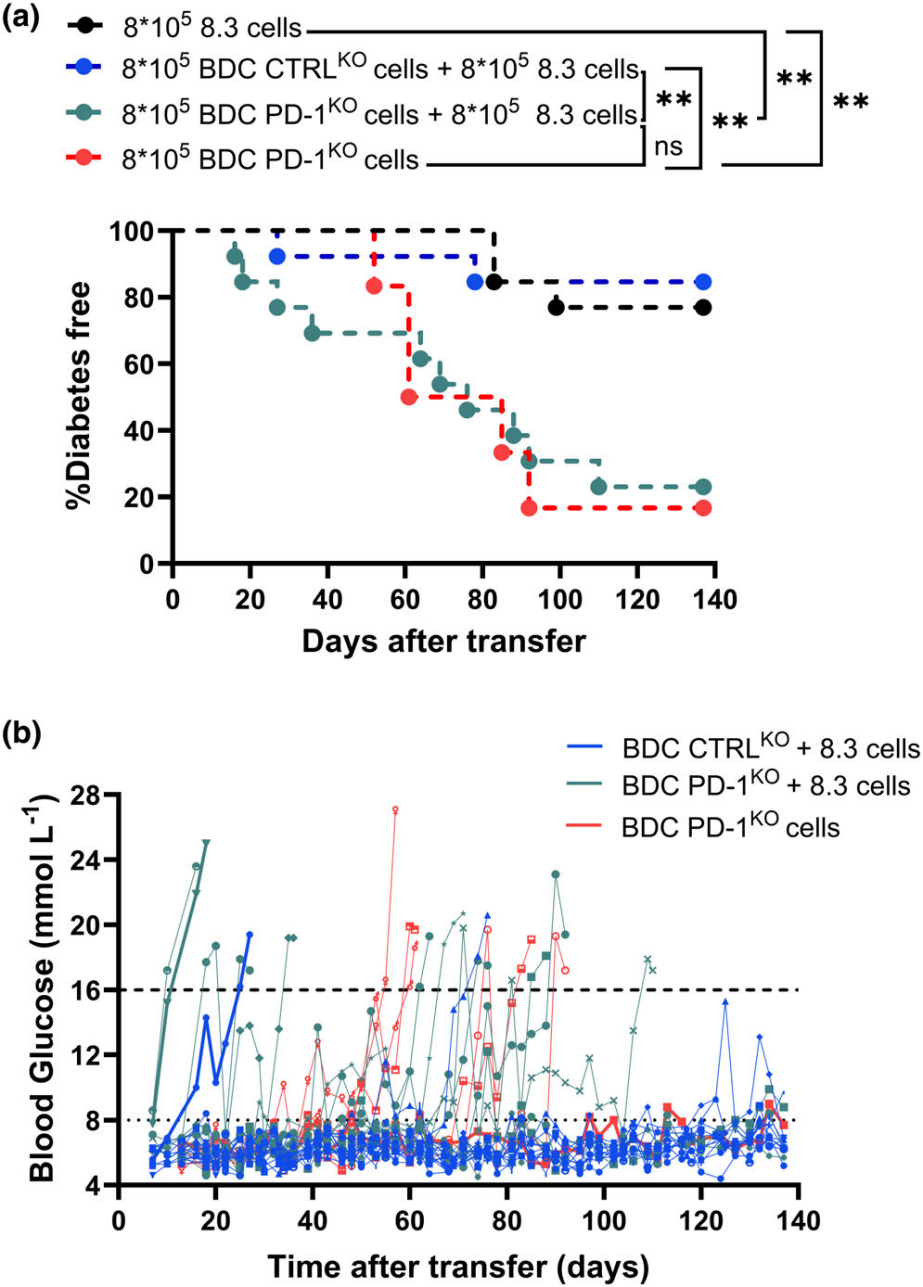
Lack of PD‐1 in BDC2.5 cells accelerates diabetes progression in NOD mice. Female NOD mice (9–11 weeks of age) received purified 8.3 cells alone (black), or with purified BDC2.5 CTRL^KO^ (blue) or PD‐1^KO^ (green) cells. Blood glucose was measured every other day until diabetes was confirmed, after two readings >16 mmol L^−1^. **(a)** Diabetes incidence and **(b)** blood glucose traces of individual mice. Dotted line represents normoglycemic threshold. Dashed line represents diabetes threshold. Data were pooled from 2 experiments; each dot represents a mouse (*n* = 13 per group), BDC PD‐1^KO^ cells group (*n* = 6 per group). The Cox–Mantel log‐rank test was performed between the survival curves. *P‐value < 0.05, **P‐value < 0.005. One mouse from BDC CTRL^KO^ + 8.3 cells group developed arthritis spontaneously and was sacrificed at day 69, mouse was normoglycemic.

Given the loss of bystander tolerance of 8.3 and other CD8^+^ T cells within the pancreas, we investigated whether the loss of PD‐1 in BDC2.5 CD4^+^ T cells impacted diabetes progression. We transferred BDC2.5/CTRL^KO^ or BDC2.5/PD‐1^KO^ CD4^+^ T cells together with 8.3 cells into NOD mice and monitored them for diabetes. Some mice were culled 7 days post‐transfer to investigate pancreas histology (Supplementary figure 7a). At this early timepoint, the level of infiltration was similar between the groups. For diabetes monitoring, we included a control 8.3 cell‐only group and a BDC2.5 PD‐1^KO^ cell‐only group. Mice that received BDC2.5 PD‐1^KO^ cells, with or without 8.3 cell transfer, developed diabetes at an accelerated rate compared to the other groups (Figure 7a). Although diabetes started earlier in the mice cotransferred with 8.3 T cells, the overall survival did not differ. A distinct fluctuation in blood glucose levels was observed when mice received PD‐1 deficient BDC2.5 cells (Figure 7b). This confirms loss of PD‐1 in BDC2.5 CD4^+^ T cells is sufficient to break tolerance and provokes accelerated autoimmunity in NOD mice. Transfer of 0.8 million naïve 8.3 cells alone did not accelerate diabetes, consistent with prior studies showing that 8.3 cells need to be activated to transfer diabetes.^31^ PD‐1 expression remained low in BDC2.5/PD‐1^KO^ cells located in the pancreas, twenty weeks after transfer (Supplementary figure 7b). In the other tissues, there were very low proportions or no BDC2.5 cells at this timepoint. Overall, the absence of PD‐1 in a small proportion of CD4^+^ T cells can greatly impact inflammation and autoimmunity in NOD mice.

## DISCUSSION

PD‐1 is a widely studied inhibitory molecule, currently targeted in the clinic for cancer immunotherapy. The involvement of PD‐1 in maintaining bystander tolerance has not yet been dissected, which is of relevance for immunotherapy design for autoimmune diseases. Here, we studied the role of PD‐1 expression in CD4^+^ T cells during bystander tolerance of islet‐specific CD8^+^ T cells in a model of T1D. We found that CRISPR‐mediated deletion of PD‐1 increased the overall proliferation of chromogranin A‐specific BDC2.5 CD4^+^ T cells that acquired a T‐bet^+^ ICOS^+^ CD107a^+^ IFN‐γ^+^ effector phenotype in the pLN and subsequently infiltrated the pancreas. Lack of PD‐1 in BDC2.5 T cells enhanced the proliferation of IGRP‐specific 8.3 CD8^+^ T cells exclusively in the pLN. These 8.3 cells developed an ICOS^+^ phenotype with increased degranulation potential in the pancreas. The BDC2.5 cells needed to exit the pLN and enter the pancreas for the enhanced activation of 8.3 cells to occur, suggesting an indirect mechanism not a local effect within the pLN. The loss of PD‐1 expression from a small population of BDC2.5 cells alone was sufficient to accelerate diabetes onset in NOD mice. Together, these results show that PD‐1 in CD4^+^ T cells is essential for bystander tolerance to CD8^+^ T cells and aids in the maintenance of peripheral tolerance.

In autoimmune settings, PD‐1^+^ CD4^+^ T cells play a critical role in controlling immune responses, in line with our observation of higher proliferation and increased numbers of BDC2.5/PD‐1^KO^ cells. In our conditional knockout model, BDC2.5/PD‐1^KO^ cells located in the pLN expressed ICOS, T‐bet, CTLA‐4 and c‐MAF^lo^ consistent with ICOS^hi^ T‐bet^hi^ terminally differentiated Th1 cells found after α‐PD‐1, α‐PD‐L1 or α‐CTLA‐4 therapy^32^, ^33^, ^34^; or increased expression of c‐MAF, RORC2 and T‐bet in Th17 cells following ICOS ligation.^35^, ^36^ The effector potential of these cells was further confirmed *ex vivo*, where stimulated BDC2.5/PD‐1^KO^ cells reduced IL‐10 expression in the spleen and increased CD107a and IFN‐γ expression in pLN and pancreas. While IL‐17 expression in the spleen showed no changes four days post‐transfer, it is still possible for BDC2.5/PD‐1^KO^ cells to further differentiate into Th17 cells. Earlier studies blocking the PD‐1/PD‐L1 pathway abrogated tolerance by preventing TCR‐driven stop signals and prolonging dendritic cell interactions that augmented TCR signaling.^37^ This prolonged T‐cell–DC interaction might explain the commitment of BDC2.5/PD‐1^KO^ CD4^+^ T cells to differentiate into Th1 cells,^34^, ^38^ similar to cells found in pancreatic islet transplants that suffer rejection.^39^, ^40^ In a chronic infection model, PD‐1 blockade promoted Th1 cytokine production and acquisition of cytotoxic killing function by viral‐specific CD4^+^ T cells.^34^ Thus, the PD‐1^KO^ BDC2.5 cells likely promoted beta cell killing in the pancreas, potentially including via direct cytotoxic effects. Overall, effector phenotypes in the pLN and their increased pancreatic infiltration suggest a pathogenic role for BDC2.5/PD1^KO^ cells which was supported by increased progression to diabetes.

Our model of conditional PD‐1^KO^, supports that BDC2.5/PD‐1^KO^ CD4^+^ T cells are likely responsible for boosting CD8^+^ T‐cell proliferation and activation. IGRP‐specific CD8^+^ T cells upregulated ICOS and degranulation activity, suggesting greater differentiation into Teff cells.^41^ This break in tolerance was supported by signs of epitope spreading, as endogenous pancreatic CD8^+^ T cells produced granzyme B in the presence of BDC2.5/PD‐1^KO^ CD4^+^ T cells. It is well established that CD4^+^ T cells provide help and enhance both the primary expansion of CD8^+^ T cells and the development of a CD8^+^ T‐cell memory phenotype.^26^, ^42^, ^43^ CD4^+^ T‐cell help classically involves CD40‐CD40L signaling to “license” DC that present both the CD4 and CD8 T‐cell epitopes on the same cell.^44^ DC licensing leads to a direct modification of the DC via cell–cell contact between MHC‐II and TCR.^43^ As CD40 was not upregulated on DC in the pLN, and FTY720 treatment prevented the CD8^+^ T‐cell proliferation boost, it is unlikely that PD‐1^KO^ BDC2.5 cells provided T‐cell help via licensing of DC locally in the pLN. However, we cannot discard that licensing of the CD8^+^ T cells happens directly in the pancreas by local activated professional APCs.

A recent study in infection models showed that both high antigen load and inflammation are required to break tolerance.^45^ Similarly, we found that the increased bystander activation of 8.3 cells was prevented by FTY720 treatment. This supports that BDC2.5/PD‐1^KO^ CD4^+^ T cell need to infiltrate the pancreas first causing islet damage, which potentially releases additional islet antigens and recruits APCs capable of presenting antigens to both CD4^+^ and CD8^+^ T cells. Others have shown that beta cells can upregulate MHC‐II in the presence of IFN‐γ; therefore, PD‐1^KO^ BDC2.5 cells may be able to directly kill islets facilitating this process.^46^ Another explanation could be the enhanced antigen presentation ability of APCs due to the inflammatory environment promoted by the BDC2.5/PD‐1^KO^ cells. Here we showed that the lack of PD‐1 in a small proportion of BDC2.5 cells was sufficient to accelerate disease after transfer into intact NOD mice. This demonstrates how accelerated tissue damage by BDC2.5 cells can enhance the activation of other islet‐specific CD8^+^ T cells, promoting T1D. Our findings have implications for immune‐related adverse events following single or combined checkpoint inhibition,^47^ an important consideration in the management of oncological patients.^48^

Loss of PD‐1 from BDC2.5 cells did not impact the proportion of Foxp3^+^ BDC2.5 cells. Loss of PD‐1 from Foxp3^+^ Tregs has been shown to increase their suppressive activity by removal of a restraint mechanism, as well as giving them a survival advantage.^49^ Specific ablation of PD‐1 from Foxp3^+^ Tregs was shown to protect from diabetes in NOD mice.^49^ In our model, the number of Tregs transferred might not have been enough to arrest proliferation and activation of PD‐1^KO^ cells. Therefore, it is unlikely that the enhanced CD8^+^ T‐cell activation we observed was due to disruption of Foxp3^+^ Treg suppression.

A limitation of our study was that the conditional knockout was not 100% efficient. In particular, full loss of PD‐1 expression required several cell divisions likely due to the time to turnover PD‐1 protein already present on the cells. We also could not exclude CRISPR off‐target effects were present. However, the CRISPR system was a useful approach for rapid deletion of genes of interest in particular on the NOD background. Some discrepancies were seen in absolute numbers in BDC2.5 cells in the spleen between experiments, which may have been due to variability in the age of the mice and the degree of pre‐existing insulitis. Our FTY720 migration assay resulted in lower viability and total numbers of endogenous cells, which might have provided a favorable environment for the accumulation of the transferred cells. It remains to be seen whether APCs or islets have enhanced antigen presentation ability in this model.

In conclusion, using conditional knockout models we established that PD‐1 expression by BDC2.5 T cells is required to maintain bystander tolerance of IGRP cells as well as restraining the CD4^+^ T cells themselves, preventing their acquisition of effector function. Thus, loss of PD‐1 may promote epitope spreading and drive acquisition of effector function in other T cells, confirming its crucial role in sustaining peripheral tolerance. The findings and model used in this study could be used to understand how to enhance bystander tolerance mechanisms that will potentially increase disease protection and translation of immunotherapies to humans.

## METHODS

### Mice

Nonobese diabetic (*NOD/ShiLtJArc*) mice (referred to as NOD mice) were purchased from the Animal Resources Centre (ARC, Perth WA, Australia). NOD BDC2.5 TCR transgenic mice (*NOD.Cg‐Tg (TcraBDC2.5 TcrbBDC2.5)1Doi/DoiJ)* ^12^ intercrossed with CD90.1 congenic NOD mice *(NOD.Cg‐Thy1a*)^50^ were used to track chromogranin A‐derived CD4^+^ T‐cell responses. NOD 8.3 TCR transgenic mice (*NOD.Cg‐Tg(TcraTcrbNY8.3)1Pesa/DvsJ*),^14^ also expressing the CD90.1 congenic marker^51^ were used to track IGRP_206‐214_ CD8^+^ T‐cell responses. Transgenic mice were originally obtained from Jackson Laboratories (Bar Harbor, ME, USA) and were bred under specific pathogen‐free conditions at the Translational Research Institute animal facility. All procedures were approved by the animal ethics committee of the University of Queensland. NOD and NOD 8.3 mice were 8–12 weeks of age; NOD BDC2.5 mice were 10–20 weeks of age. All mice used were females.

### T‐cell isolation and labeling

Spleen and lymph nodes, except pancreatic LN (pLN), from NOD BDC2.5 and NOD 8.3 mice were collected in MACS buffer (Miltenyi Biotec, Bergisch‐Gladbach, Germany) and processed into single‐cell suspensions. T cells were isolated using the CD4^+^ T‐cell isolation kit (Miltenyi Biotec) or the CD8^+^ T‐cell isolation kit (Miltenyi Biotec), respectively, using an autoMACS according to the manufacturer's instructions. Purified BDC2.5 or 8.3 cells were then labeled with Cell Trace violet (CTV) (Invitrogen, Waltham, MA, USA) or Cell trace Carboxyfluorescein succinimidyl ester (CFSE) (Invitrogen) according to the manufacturer's instructions.

### CRISPR/Cas9 knockout

Eight to ten million purified BDC2.5 cells were resuspended in PBS and aliquoted for CRISPR/Cas9 targeting reaction. Cells were electroporated with sgRNA‐CRISPR/Cas9 using the following sgRNAs: nontargeted control sgRNA 5′‐GCACUACCAGAGCUAACUCA‐3′ or PD‐1 specific sgRNA 5′‐GACACACGGCGCAAUGACAG‐3′ (Synthego, Redwood City, CA, USA), as previously described.^16^ Briefly, a mix of P3 buffer (Lonza, Basel, Switzerland), Cas9 (Integrated DNA Technologies, Coralville, IA, USA) and sgRNA were added to cells and electroporated in the Lonza Nucleofector X unit using the “T cell mouse unstim” program. Cells were resuspended in complete RPMI and rested for 10 min at 37°C for viability and recovery purposes. Cells were then washed in dPBS (Invitrogen) and injected intravenously (0.8 million cells of BDC2.5 and/or 8.3 cells) per mouse for adoptive transfer.

For lymphocyte migration assays, NOD mice were treated with 3 mg kg^−1^ of FTY720 (Sigma‐Aldrich, St Louis MI, USA) or water by intraperitoneal injection 1 day before and 1 day after the adoptive transfer of electroporated‐BDC2.5 and 8.3 cells. Mice that still had BDC2.5 cell infiltration into the pancreas were excluded from the analysis.

### Tissue digestion and cell processing

Spleen, pLN and inguinal lymph nodes (iLN) were collected in FACS buffer consisting of PBS containing 20 mM EDTA (Sigma‐Aldrich) and 0.05% bovine serum albumin (BovoGEN, Melbourne VIC, Australia). For antigen‐presenting cell isolation and pancreata, samples were digested with collagenase III (Worthington Biochem, Lakewood NJ, USA) and DNase I (Sigma‐Aldrich) for 20 min at 37°C. Single‐cell suspensions were made via dispersion through a cell strainer. For pancreas, single‐cell suspensions were overlaid onto a histopaque solution (Sigma) and separated by density‐centrifugation. The interface was collected for flow cytometry staining. Spleen samples were treated with ACK lysis buffer (Thermo Fisher Scientific, Waltham, MA, USA) to lyse red blood cells. For cytokine measurements, cell suspensions were pre‐treated with PMA/ionomycin cell stimulation cocktail (BioLegend, San Diego, CA, USA) for 30 minutes following the addition of CD107a‐AF647 (1DB4) (BioLegend) antibody together with monensin (Invitrogen) and brefeldin A (BioLegend) for an additional 3.5 h prior to flow cytometry staining.

### Flow cytometry

Cells were washed with PBS and stained with Live/Dead fixable aqua dead cell stain kit (Thermo Fischer Scientific) or Fixable viability stain 440 (BD Horizon, BD Biosciences, Franklin Lakes, NJ, USA) for 15 min at room temperature. Cells were washed with FACS buffer and then stained with surface antibodies for 20 min at 4°C. For T‐cell phenotyping, antibodies were as follows: CD90.1‐Percp‐cy5.5 (OX‐7), CD69‐APC‐efluor 780 (H1.2F3), ICOS‐FITC (C398.4A), PD‐1‐PE‐cy7 (29F.1A12), DNAM‐1‐BV650 (TX42.1), CD4‐BUV496 (GK1.5), CD8‐BV711 (53–6.7), CD44‐BUV395 (IM7), CD49b‐BV786 (HMα2), TIGIT‐BV421 (1G9) (BD Biosciences) and CD62L‐PE (MEL‐14) (Invitrogen). For antigen‐presenting cell phenotyping, a murine flow panel was adapted from Rodahl *et al*.,^22^ mAb included: F4/80‐PE‐cy5 (BM8), CD40‐PE‐cy7 (3123), CD11c‐BV605 (N418), CD19‐BV570 (6D5), Ly6G‐Percp‐cy5.5 (1A8), TCRbeta‐Percp‐cy5.5 (H57‐597), XCR1‐BV650 (ZET), PDCA‐1‐PE (927), MHC‐II‐AF647 (39‐10‐8), PD‐L1‐PE‐Dazzle594 (10F.9G2), H‐2K^d^‐FITC (SF1‐1.1) from BioLegend; CD11b‐BUV395 (M1/70), CD155‐BV421 (TX56), CD86‐BUV563 (PO3), CCR7‐BUV737 (4B12) and CD112‐BV786 (829038) from BD Biosciences and Ly6c‐APC‐eflour780 (HK1.4) from Invitrogen. Antigen‐presenting cells were washed and fixed with 4% paraformaldehyde (PFA) (ProSciTech). For T‐cell phenotyping, cells were permeabilized and fixed using Foxp3/Transcription Factor Staining Buffer Set (eBioscience, Thermo Fisher Scientific) or with Cytofix/Cytoperm kit (BD Bioscience) for cytokine staining. Intracellular staining antibodies included the following: IL‐10‐PE (JES5‐16E3), Granzyme B‐PE‐Dazzle594 (QA16A02) IFN‐γ‐AF488 (XGM1.2), T‐bet‐AF647 (4B10) (BioLegend), CTLA‐4‐APCR700 (UC10‐4B9) (BD Biosciences), TNF‐PE‐cy7 (MP6‐XT22), Foxp3‐PE‐cy5 (FJK‐16 s), cMAF‐PE (Sym0F1), EOMES‐PE‐cy7 (Dan11mag) (Invitrogen). Cells were washed and counting beads (Beckman Coulter, Brea CA, USA) added before samples were acquired in a LSR Fortessa X20 (BD Bioscience) or a BD FACS Symphony A5 (BD Bioscience) using FACS DiVa 8.0.1 software.

Data were analyzed using FlowJo V10.8.1 software (Tree Star, Ashland, OR, USA). The gating strategy is shown in Supplementary figure 8. Data were gated using time, lymphocytes, doublet and dead cell exclusion. Fluorescence‐minus‐one controls were used to determine background staining. For clustering analysis, samples were compensated and CD4^+^ CD90.1^+^ or CD8^+^ CD90.1^+^ T cells were concatenated from all samples and all tissues into a file. Concatenated files were analyzed using flow analysis by self‐organizing maps using FlowSOM v2.6 clustering using a grid size 10 × 10, cluster explorer and t‐distributed stochastic neighbor embedding (t‐SNE) using perplexity (p) and iterations (i) for BDC2.5 cells (p:800 i:80) and 8.3 cells (p:800 i:15). All channels were included in the clustering algorithms except from PD‐1 in BDC2.5, to avoid clustering bias.

### Histology

Pancreata were fixed in 10% neutral buffered formalin (Sigma‐Aldrich) and stained using hematoxylin and eosin. Slides were scanned using an Olympus VS120 microscope at 20× magnification.

### Diabetes incidence

Blood glucose was monitored every other day, starting at 15 days postadoptive transfer using a FreeStyle Libre 2 blood glucose reader (Abbott, Chicago, IL, USA). Mice were diagnosed as diabetic after two confirmed readings of 16 mmol L^−1^ or above.

### Statistical analysis

Data were analyzed using GraphPad Prism Version 8.01 software (GraphPad, La Jolla, CA, USA). Data are presented as mean ± SEM. For each experiment, the number of replicates and statistical analyses are mentioned in the legend. For tests in individual tissues, one‐way analysis of variance (ANOVA) and Tukey's multiple comparison test was performed. For coexpression of proteins on the same tissue, a two‐way ANOVA and Sidak's or Tukey's multiple comparison test were performed when analyzing transgenic or endogenous cells, respectively. Two‐way comparisons used an unpaired two‐tailed *t‐*test with Welch's correction. For comparing survival curves in diabetes incidence experiments, a Cox–Mantel log‐rank test was performed. Statistical significance was considered to be *P‐value < 0.05, **P‐value < 0.005, ***P‐value < 0.0005, ****P‐value < 0.0001.

## AUTHOR CONTRIBUTIONS

**Jeniffer D Loaiza Naranjo:** Conceptualization; data curation; formal analysis; investigation; methodology; visualization; writing – original draft. **Vivian Zhang:** Investigation; writing – review and editing. **Rathna Ravichandran:** Formal analysis; investigation. **Anne‐Sophie Bergot:** Supervision; writing – review and editing. **Ranjeny Thomas:** Conceptualization; funding acquisition; resources; supervision; writing – review and editing. **Emma E Hamilton‐Williams:** Conceptualization; funding acquisition; project administration; resources; supervision; writing – original draft.

## CONFLICT OF INTEREST

The authors declare no conflict of interest.

## Supporting information


Supplementary figure 1.
**Supplementary figure 2**.
**Supplementary figure 3**.
**Supplementary figure 4**.
**Supplementary figure 5**.
**Supplementary figure 6**.
**Supplementary figure 7**.
**Supplementary figure 8**.

## Data Availability

The data that support the findings of this study are available from the corresponding author upon reasonable request.

## References

[1] Kent SC , Mannering SI , Michels AW , Babon JAB . Deciphering the pathogenesis of human type 1 diabetes (T1D) by interrogating T cells from the “scene of the crime”. Curr Diab Rep 2017; 17: 95. 28864875 10.1007/s11892-017-0915-y PMC5600889

[2] Hamilton‐Williams EE , Bergot AS , Reeves PL , Steptoe RJ . Maintenance of peripheral tolerance to islet antigens. J Autoimmun 2016; 72: 118–125. 27255733 10.1016/j.jaut.2016.05.009

[3] Ansari MJ , Salama AD , Chitnis T , *et al*. The programmed death‐1 (PD‐1) pathway regulates autoimmune diabetes in nonobese diabetic (NOD) mice. J Exp Med 2003; 198: 63–69. 12847137 10.1084/jem.20022125 PMC2196083

[4] Fife BT , Guleria I , Gubbels Bupp M , *et al*. Insulin‐induced remission in new‐onset NOD mice is maintained by the PD‐1‐PD‐L1 pathway. J Exp Med 2006; 203: 2737–2747. 17116737 10.1084/jem.20061577 PMC2118162

[5] Bergot AS , Buckle I , Cikaluru S , *et al*. Regulatory T cells induced by single‐peptide liposome immunotherapy suppress islet‐specific T cell responses to multiple antigens and protect from autoimmune diabetes. J Immunol 2020; 204: 1787–1797. 32111734 10.4049/jimmunol.1901128 PMC9352518

[6] Ogando J , Sáez ME , Santos J , *et al*. PD‐1 signaling affects cristae morphology and leads to mitochondrial dysfunction in human CD8^+^ T lymphocytes. J Immunother Cancer 2019; 7: 151. 31196176 10.1186/s40425-019-0628-7 PMC6567413

[7] Damo M , Hornick NI , Venkat A , *et al*. PD‐1 maintains CD8 T cell tolerance towards cutaneous neoantigens. Nature 2023; 619: 151–159. 37344588 10.1038/s41586-023-06217-y PMC10989189

[8] Vecchione A , Di Fonte R , Gerosa J , *et al*. Reduced PD‐1 expression on circulating follicular and conventional FOXP3^+^ Treg cells in children with new onset type 1 diabetes and autoantibody‐positive at‐risk children. Clin Immunol 2020; 211: 108319. 31794865 10.1016/j.clim.2019.108319

[9] Ai L , Xu A , Xu J . Roles of PD‐1/PD‐L1 pathway: signaling, cancer, and beyond. Adv Exp Med Biol 2020; 1248: 33–59. 32185706 10.1007/978-981-15-3266-5_3

[10] Pauken KE , Torchia JA , Chaudhri A , Sharpe AH , Freeman GJ . Emerging concepts in PD‐1 checkpoint biology. Semin Immunol 2021; 52: 101480. 34006473 10.1016/j.smim.2021.101480 PMC8545711

[11] Delong T , Wiles TA , Baker RL , *et al*. Pathogenic CD4 T cells in type 1 diabetes recognize epitopes formed by peptide fusion. Science 2016; 351: 711–714. 26912858 10.1126/science.aad2791 PMC4884646

[12] Katz JD , Wang B , Haskins K , Benoist C , Mathis D . Following a diabetogenic T cell from genesis through pathogenesis. Cell 1993; 74: 1089–1100. 8402882 10.1016/0092-8674(93)90730-e

[13] Stadinski BD , Delong T , Reisdorph N , *et al*. Chromogranin a is an autoantigen in type 1 diabetes. Nat Immunol 2010; 11: 225–231. 20139986 10.1038/ni.1844 PMC3166626

[14] Verdaguer J , Schmidt D , Amrani A , Anderson B , Averill N , Santamaria P . Spontaneous autoimmune diabetes in monoclonal T cell nonobese diabetic mice. J Exp Med 1997; 186: 1663–1676. 9362527 10.1084/jem.186.10.1663 PMC2199139

[15] Krishnamurthy B , Dudek NL , McKenzie MD , *et al*. Responses against islet antigens in NOD mice are prevented by tolerance to proinsulin but not IGRP. J Clin Invest 2006; 116: 3258–3265. 17143333 10.1172/JCI29602 PMC1679712

[16] Nüssing S , House IG , Kearney CJ , *et al*. Efficient CRISPR/Cas9 gene editing in uncultured naive mouse T cells for in vivo studies. J Immunol 2020; 204: 2308–2315. 32152070 10.4049/jimmunol.1901396

[17] Soghoian DZ , Jessen H , Flanders M , *et al*. HIV‐specific cytolytic CD4 T cell responses during acute HIV infection predict disease outcome. Sci Transl Med 2012; 4: 123ra125. 10.1126/scitranslmed.3003165 PMC3918726 22378925

[18] Umeshappa CS , Mbongue J , Singha S , *et al*. Ubiquitous antigen‐specific T regulatory type 1 cells variably suppress hepatic and extrahepatic autoimmunity. J Clin Invest 2020; 130: 1823–1829. 32125290 10.1172/JCI130670 PMC7108901

[19] Ahlers J , Mantei A , Lozza L , *et al*. A notch/STAT3‐driven Blimp‐1/c‐Maf‐dependent molecular switch induces IL‐10 expression in human CD4^+^ T cells and is defective in Crohn's disease patients. Mucosal Immunol 2022; 15: 480–490. 35169232 10.1038/s41385-022-00487-x PMC9038525

[20] Chikuma S , Terawaki S , Hayashi T , *et al*. PD‐1‐mediated suppression of IL‐2 production induces CD8^+^ T cell Anergy in vivo. J Immunol 2009; 182: 6682–6689. 19454662 10.4049/jimmunol.0900080

[21] Dong C , Juedes AE , Temann UA , *et al*. ICOS co‐stimulatory receptor is essential for T‐cell activation and function. Nature 2001; 409: 97–101. 11343121 10.1038/35051100

[22] Rodahl I , Gotley J , Andersen SB , *et al*. Acquisition of murine splenic myeloid cells for protein and gene expression profiling by advanced flow cytometry and CITE‐seq. STAR Protoc 2021; 2: 100842. 34585169 10.1016/j.xpro.2021.100842 PMC8456112

[23] Ferris ST , Carrero JA , Mohan JF , Calderon B , Murphy KM , Unanue ER . A minor subset of Batf3‐dependent antigen‐presenting cells in islets of Langerhans is essential for the development of autoimmune diabetes. Immunity 2014; 41: 657–669. 25367577 10.1016/j.immuni.2014.09.012 PMC4220295

[24] Serreze DV , Gaskins HR , Leiter EH . Defects in the differentiation and function of antigen presenting cells in NOD/Lt mice. J Immunol 1993; 150: 2534–2543. 8450229

[25] Nikolic T , Woittiez NJC , Van Der Slik A , *et al*. Differential transcriptome of tolerogenic versus inflammatory dendritic cells points to modulated T1D genetic risk and enriched immune regulation. Genes Immun 2017; 18: 176–183. 28794505 10.1038/gene.2017.18

[26] Kurts C , Carbone FR , Barnden M , *et al*. CD4^+^ T cell help impairs CD8^+^ T cell deletion induced by cross‐presentation of self‐antigens and favors autoimmunity. J Exp Med 1997; 186: 2057–2062. 9396776 10.1084/jem.186.12.2057 PMC2199175

[27] Belz GT , Behrens GM , Smith CM , *et al*. The CD8alpha^+^ dendritic cell is responsible for inducing peripheral self‐tolerance to tissue‐associated antigens. J Exp Med 2002; 196: 1099–1104. 12391021 10.1084/jem.20020861 PMC2194045

[28] Huwiler A , Pfeilschifter J . New players on the center stage: sphingosine 1‐phosphate and its receptors as drug targets. Biochem Pharmacol 2008; 75: 1893–1900. 18321471 10.1016/j.bcp.2007.12.018

[29] Dixit D , Hallisey VM , Zhu EYS , *et al*. S1PR1 inhibition induces proapoptotic signaling in T cells and limits humoral responses within lymph nodes. J Clin Invest 2024; 134: e174984. 38194271 10.1172/JCI174984 PMC10869180

[30] Kendall PL , Yu G , Woodward EJ , Thomas JW . Tertiary lymphoid structures in the pancreas promote selection of B lymphocytes in autoimmune diabetes. J Immunol 2007; 178: 5643–5651. 17442947 10.4049/jimmunol.178.9.5643

[31] Manirarora JN , Wei CH . Combination therapy using IL‐2/IL‐2 monoclonal antibody complexes, rapamycin, and islet autoantigen peptides increases regulatory T cell frequency and protects against spontaneous and induced type 1 diabetes in nonobese diabetic mice. J Immunol 2015; 195: 5203–5214. 26482409 10.4049/jimmunol.1402540

[32] Chen H , Fu T , Suh WK , *et al*. CD4 T cells require ICOS‐mediated PI3K signaling to increase T‐bet expression in the setting of anti‐CTLA‐4 therapy. Cancer Immunol Res 2014; 2: 167–176. 24778280 10.1158/2326-6066.CIR-13-0155 PMC4004958

[33] Wei SC , Levine JH , Cogdill AP , *et al*. Distinct cellular mechanisms underlie anti‐CTLA‐4 and anti‐PD‐1 checkpoint blockade. Cell 2017; 170: 1120–1133.e1117. 28803728 10.1016/j.cell.2017.07.024 PMC5591072

[34] Snell LM , Xu W , Abd‐Rabbo D , *et al*. Dynamic CD4^+^ T cell heterogeneity defines subset‐specific suppression and PD‐L1‐blockade‐driven functional restoration in chronic infection. Nat Immunol 2021; 22: 1524–1537. 34795443 10.1038/s41590-021-01060-7 PMC10286806

[35] Paulos CM , Carpenito C , Plesa G , *et al*. The inducible costimulator (ICOS) is critical for the development of human T_H_17 cells. Sci Transl Med 2010; 2: 55ra78. 10.1126/scitranslmed.3000448 PMC6282816 20980695

[36] Xu J , Yang Y , Qiu G , *et al*. c‐Maf regulates IL‐10 expression during Th17 polarization. J Immunol 2009; 182: 6226–6236. 19414776 10.4049/jimmunol.0900123 PMC2834209

[37] Fife BT , Pauken KE , Eagar TN , *et al*. Interactions between PD‐1 and PD‐L1 promote tolerance by blocking the TCR‐induced stop signal. Nat Immunol 2009; 10: 1185–1192. 19783989 10.1038/ni.1790 PMC2778301

[38] Hirschhorn‐Cymerman D , Budhu S , Kitano S , *et al*. Induction of tumoricidal function in CD4^+^ T cells is associated with concomitant memory and terminally differentiated phenotype. J Exp Med 2012; 209: 2113–2126. 23008334 10.1084/jem.20120532 PMC3478933

[39] Besancon A , Demir Z , Goncalves T , *et al*. Differential impact of T‐bet and IFNγ on pancreatic islet allograft rejection. Transplantation 2018; 102: 1496–1504. 29757902 10.1097/TP.0000000000002261

[40] Ito T , Ueno T , Clarkson MR , *et al*. Analysis of the role of negative T cell costimulatory pathways in CD4 and CD8 T cell‐mediated alloimmune responses in vivo. J Immunol 2005; 174: 6648–6656. 15905503 10.4049/jimmunol.174.11.6648

[41] Rao RR , Li Q , Odunsi K , Shrikant PA . The mTOR kinase determines effector versus memory CD8^+^ T cell fate by regulating the expression of transcription factors T‐bet and Eomesodermin. Immunity 2010; 32: 67–78. 20060330 10.1016/j.immuni.2009.10.010 PMC5836496

[42] Bevan MJ . Helping the CD8^+^ T‐cell response. Nat Rev Immunol 2004; 4: 595–602. 15286726 10.1038/nri1413

[43] Smith CM , Wilson NS , Waithman J , *et al*. Cognate CD4^+^ T cell licensing of dendritic cells in CD8^+^ T cell immunity. Nat Immunol 2004; 5: 1143–1148. 15475958 10.1038/ni1129

[44] Bennett SR , Carbone FR , Karamalis F , Flavell RA , Miller JF , Heath WR . Help for cytotoxic‐T‐cell responses is mediated by CD40 signalling. Nature 1998; 393: 478–480. 9624004 10.1038/30996

[45] Van Der Byl W , Nüssing S , Peters TJ , *et al*. The CD8^+^ T cell tolerance checkpoint triggers a distinct differentiation state defined by protein translation defects. Immunity 2024; 57: 1324–1344.e1328. 38776918 10.1016/j.immuni.2024.04.026 PMC11807353

[46] Zhao Y , Scott NA , Quah HS , *et al*. Mouse pancreatic beta cells express MHC class II and stimulate CD4^+^ T cells to proliferate. Eur J Immunol 2015; 45: 2494–2503. 25959978 10.1002/eji.201445378

[47] Narumoto K , Oda N , Mitani R , Takata I . Atezolizumab‐induced type 1 diabetic ketoacidosis in a patient with small cell lung cancer and pre‐existing type 2 diabetes mellitus. Cureus 2024; 16: e57024. 38681275 10.7759/cureus.57024 PMC11046427

[48] Suijkerbuijk KPM , van Eijs MJM , van Wijk F , Eggermont AMM . Clinical and translational attributes of immune‐related adverse events. Nat Cancer 2024; 5: 557–571. 38360861 10.1038/s43018-024-00730-3

[49] Tan CL , Kuchroo JR , Sage PT , *et al*. PD‐1 restraint of regulatory T cell suppressive activity is critical for immune tolerance. J Exp Med 2021; 218: e20182232. 33045061 10.1084/jem.20182232 PMC7543091

[50] Brenu EW , Bartley TJ , Wright CM , Hamilton‐Williams EE . CD11a/ICAM‐1 blockade combined with IL‐2 targeting therapy causes a paradoxical acceleration of type 1 diabetes. Immunol Cell Biol 2017; 95: 803–813. 28611472 10.1038/icb.2017.49

[51] Hamilton‐Williams EE , Wong SBJ , Martinez X , *et al*. *Idd9.2* and *Idd9.3* protective alleles function in CD4^+^ T‐cells and nonlymphoid cells to prevent expansion of pathogenic islet‐specific CD8^+^ T‐cells. Diabetes 2010; 59: 1478–1486. 20299469 10.2337/db09-1801 PMC2874709

