# Supplementary material for: PD‐1 expressing islet‐specific CD4 + T cells promote bystander tolerance and prevent autoimmunity
Source: Immunol Cell Biol. 2025 Jul 7;103(7):738–51. doi: 10.1111/imcb.70044 (PMC12392717; doi:10.1111/imcb.70044)
Supplement: Supplementary file 1 — Supplementary figure 1. Supplementary figure 2. Supplementary figure 3. Supplementary figure 4. Supplementary figure 5. Supplementary figure 6. Supplementary figure 7. Supplementary figure 8. [file IMCB-103-738-s001.pdf]

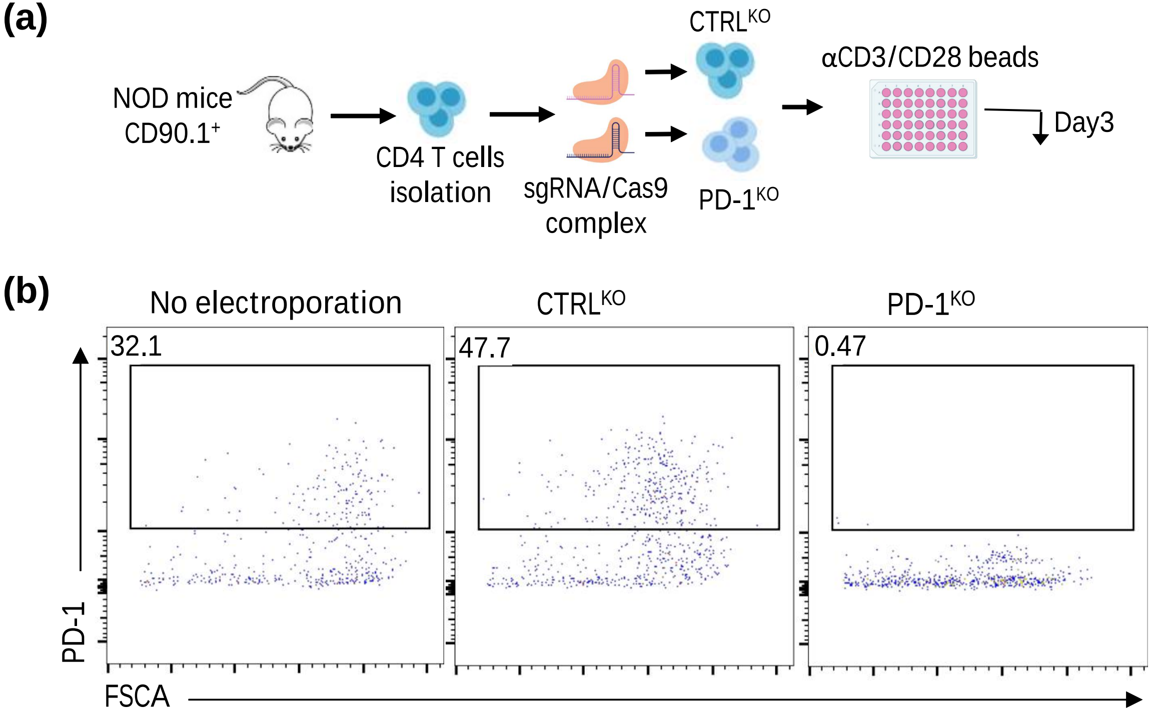

Supplementary figure 1. Validation of PD-1KO in BDC2.5 CD4^{+} T cells *in vitro* and *in vivo*. (a) Experimental design. Purified CFSE-labelled BDC2.5 cells were electroporated with untargeted CTRL or PD-1 sgRNA/Cas9. BDC2.5 cells were cultured with anti-CD3/CD28 beads for 3 days. **(b)** PD-1 expression on BDC2.5 cells 3 days post-culture. Data are representative of two experiments, wells in triplicates per group.

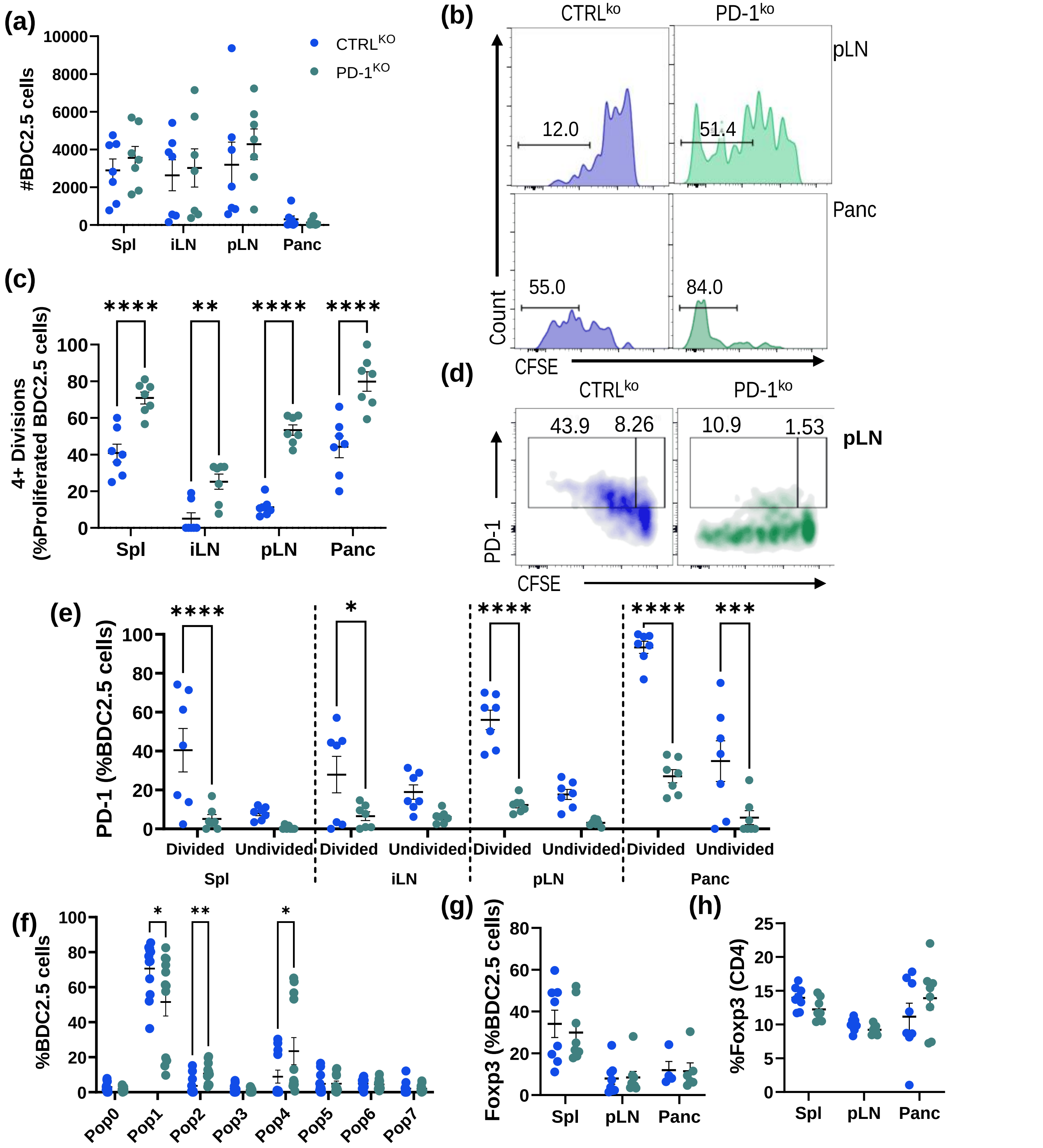

Supplementary figure 2. Lack of PD-1 expression increases the proportion of highly divided BDC2.5 cells and their differentiation, but has no impact on Foxp3$^+$ Tregs. Purified CFSE-labelled BDC2.5 cells were electroporated with untargeted CTRL (blue) or PD-1 (green) sgRNA/Cas9 and co-injected with purified CTV-labelled 8.3 cells into NOD mice. Spleen, iLN, pLN, and pancreas were collected and analysed four days after transfer. **(a)** Number of BDC2.5 cells. **(b)** Representative histograms and **(c)** proportion of proliferated BDC2.5 cells with 4+ divisions. **(d)** Representative density plots and **(e)** proportion of PD-1 in divided and undivided BDC2.5 cells. **(f)** Proportion of BDC2.5 cells in populations found after FlowSOM clustering. Proportion of Foxp3 in **(g)** BDC2.5 cells and **(h)** bulk CD4$^+$ T cells. Data are pooled from two experiments (n = 7 per group, mean ± SEM) using two-way ANOVA with Sidak's multiple comparisons test.

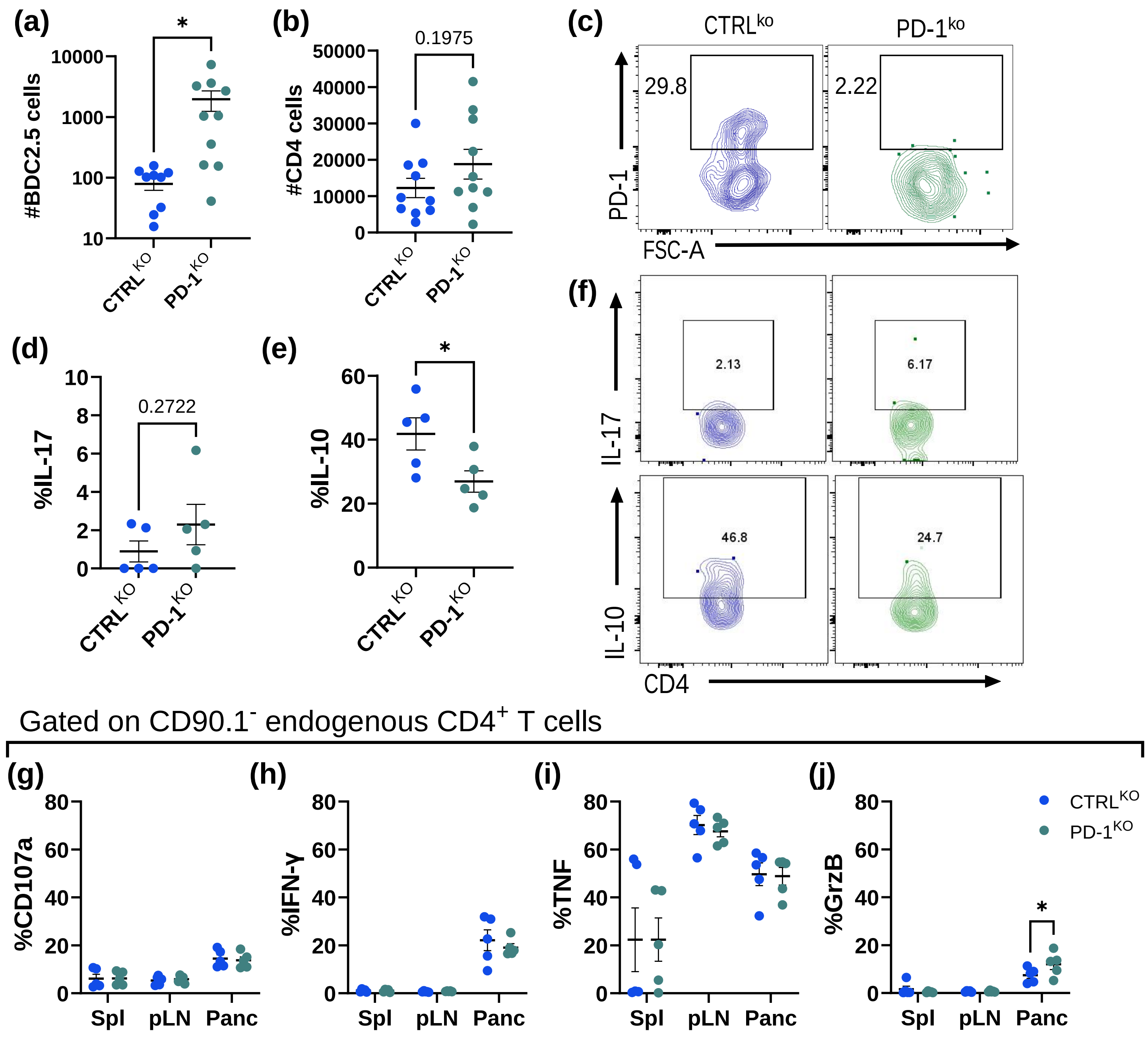

Supplementary figure 3. Lack of PD-1 expression in BDC2.5 cells reduces IL-10 production, and impacts bulk CD4$^+$ granzyme B expression. Purified CFSE-labelled BDC2.5 cells were electroporated with untargeted CTRL (blue) or PD-1 (green) sgRNA/Cas9 and co-injected with purified CTV-labelled 8.3 cells into NOD mice. Spleen, pLN, and pancreas were collected and stimulated *ex vivo* with PMA/Ionomycin. Number of **(a)** BDC2.5 cells and **(b)** CD4$^+$ T cells in the pancreas 7 days post-transfer. **(c)** Representative dot plots of PD-1 expression 7 days post-transfer. Proportion of **(d)** IL-17 and **(e)** IL-10 with **(f)** representative contour plots in splenic BDC2.5 cells 4 days post-transfer. Proportion of **(g)** CD107a, **(h)** IFN-γ, **(i)** TNF and **(j)** granzyme B in CD90.1 endogenous CD4$^+$ T cells, 7 days post-transfer. Data are from two **(a-c)**; one **(d-f)** or representative from two **(g-j)** experiment (n = 7 per group, mean ± SEM) using student's *t*-test or two-way ANOVA with Sidak's multiple comparisons test.

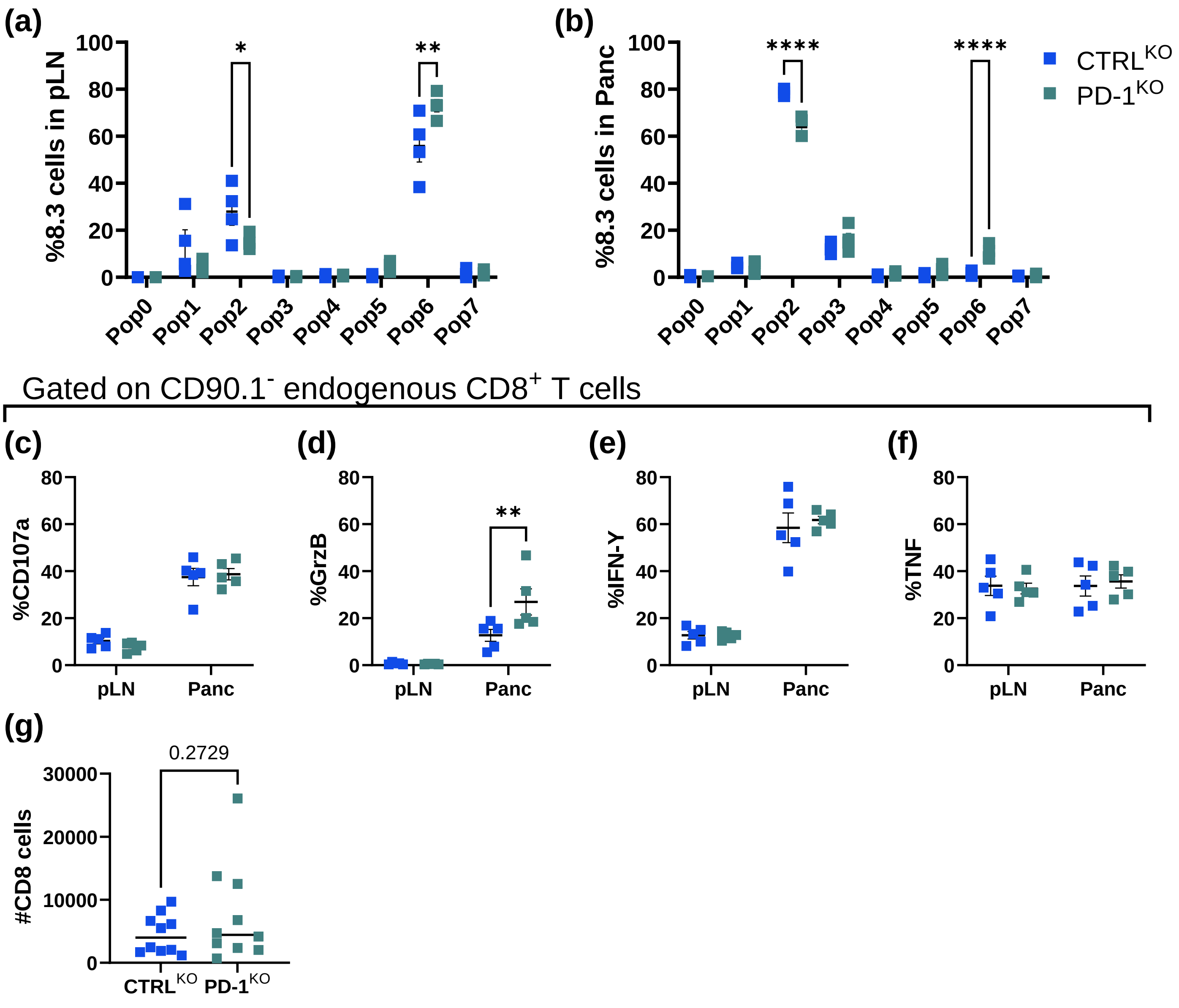

Supplementary figure 4. Transferred 8.3 CD8$^+$ T cells become activated while endogenous CD8$^+$ T cells in the pancreas become more cytotoxic in the presence of BDC2.5 PD-1^{KO} cells. Purified CFSE-labelled BDC2.5 cells were electroporated with untargeted CTRL (blue) or PD-1 (green) sgRNA/Cas9 and co-injected with purified CTV-labelled 8.3 cells into NOD mice. Proportion of 8.3 cells in populations found using FlowSOM clustering analysis in **(a)** pLN and **(b)** pancreas 4 days post-transfer. pLN and pancreas collected 7 days post-transfer were stimulated *ex vivo* with PMA/Ionomycin. Proportion of **(c)** CD107a, **(d)** Granzyme B, **(e)** IFN-y and **(j)** TNF in CD90.1 endogenous CD8+ Tcells. **(g)** Number of 8.3 cells found in pacreas 7 days post-transfer. Data are representative **(a-f)**; or pooled **(g)** from two experiments (n = 4 or 9 per group, mean ± SEM) using student's *t*-test or two-way ANOVA with Sidak's multiple comparisons test.

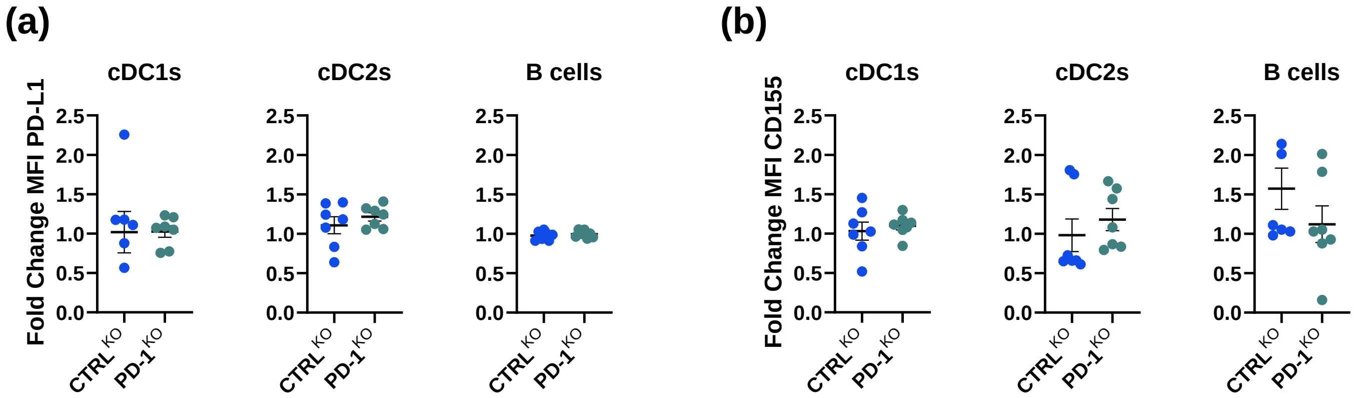

Supplementary figure 5. No change on PD-L1 or CD155 on antigen presenting cells in the presence of BDC2.5 PD-1^{KO} cells. NOD mice received purified 8.3 cells alone or together with purified BDC2.5/CTRL^{KO} (blue) or PD-1^{KO} (green) cells. pLNs were collected and analysed four days after transfer. Fold change MFI of (a) PD-L1 and (b) CD155 in cDC1s, cDC2s and B cells. Data were normalised to the 8.3 cell-only group. Data are pooled from two experiments (n = 6 or 7, mean ± SEM) and analysed using Student's *t*-test.

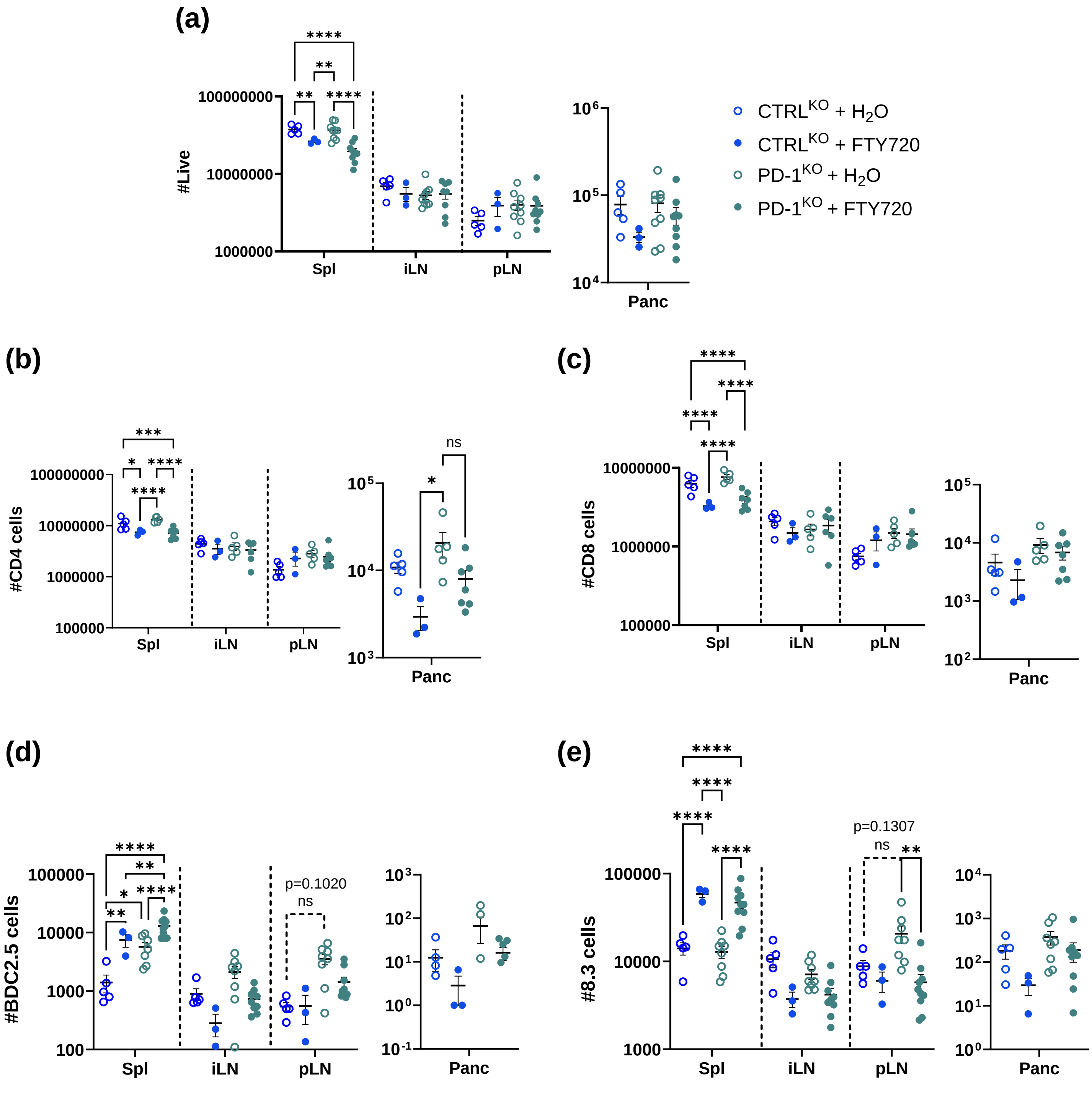

Supplementary figure 6. Number of splenic live cells and transferred T cells in the pLN are reduced in the presence of FTY720. NOD mice received CFSE-labelled BDC2.5 CTRL^{KO} (blue) or PD-1^{KO} (green) cells together with CTV-labelled 8.3 cells. Mice were treated with FTY720 or water at day-1 and day 1 (i.p), spleen, iLN, pLN and pancreas were collected at day 4. Absolute number of **(a)** live cells, **(b)** CD4^{+} Tcells, **(c)** CD8^{+} T cells, **(d)** BDC2.5 cells and **(e)** 8.3 cells. Data are pooled from two experiments (n = 3 or 7 per group, mean ± SEM) using 2-way ANOVA with Tukey's correction.

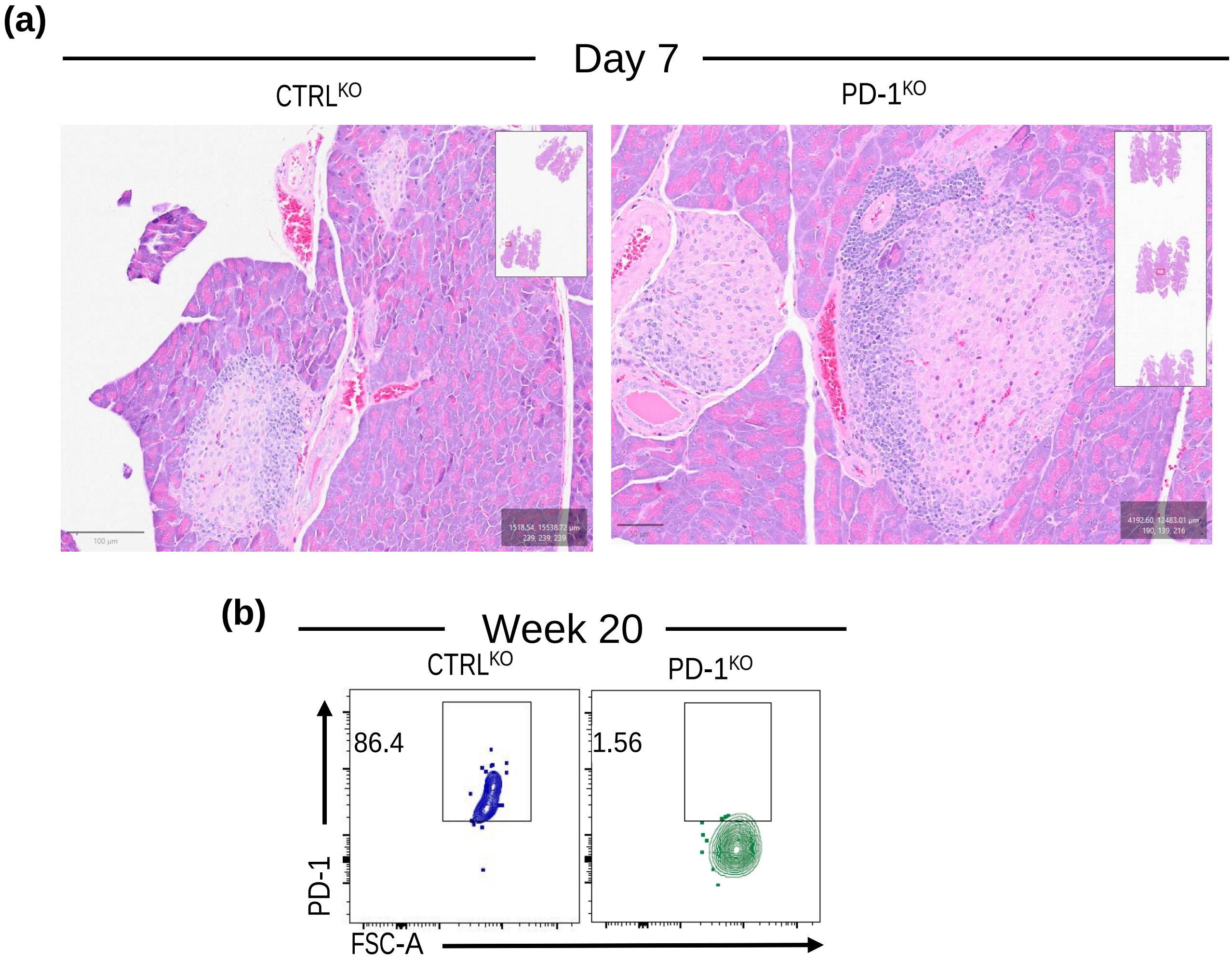

Supplementary figure 7. Expression of PD-1 does not have a significant impact on pancreas infiltration at early timepoints, but BDC2.5 cells remain in the pancreas 20-weeks post-transfer of BDC2.5 cells. NOD mice received CFSE-labelled BDC2.5 CTRL^{KO} (blue) or PD-1^{KO} (green) cells together with CTV-labelled 8.3 cells. **(a)** Representative H&E pancreata sections 7 days-post transfer. **(b)** Representative contour plots of BDC2.5 cells 20 weeks post-transfer. Scale bar 50 µm right or 100 µm left.

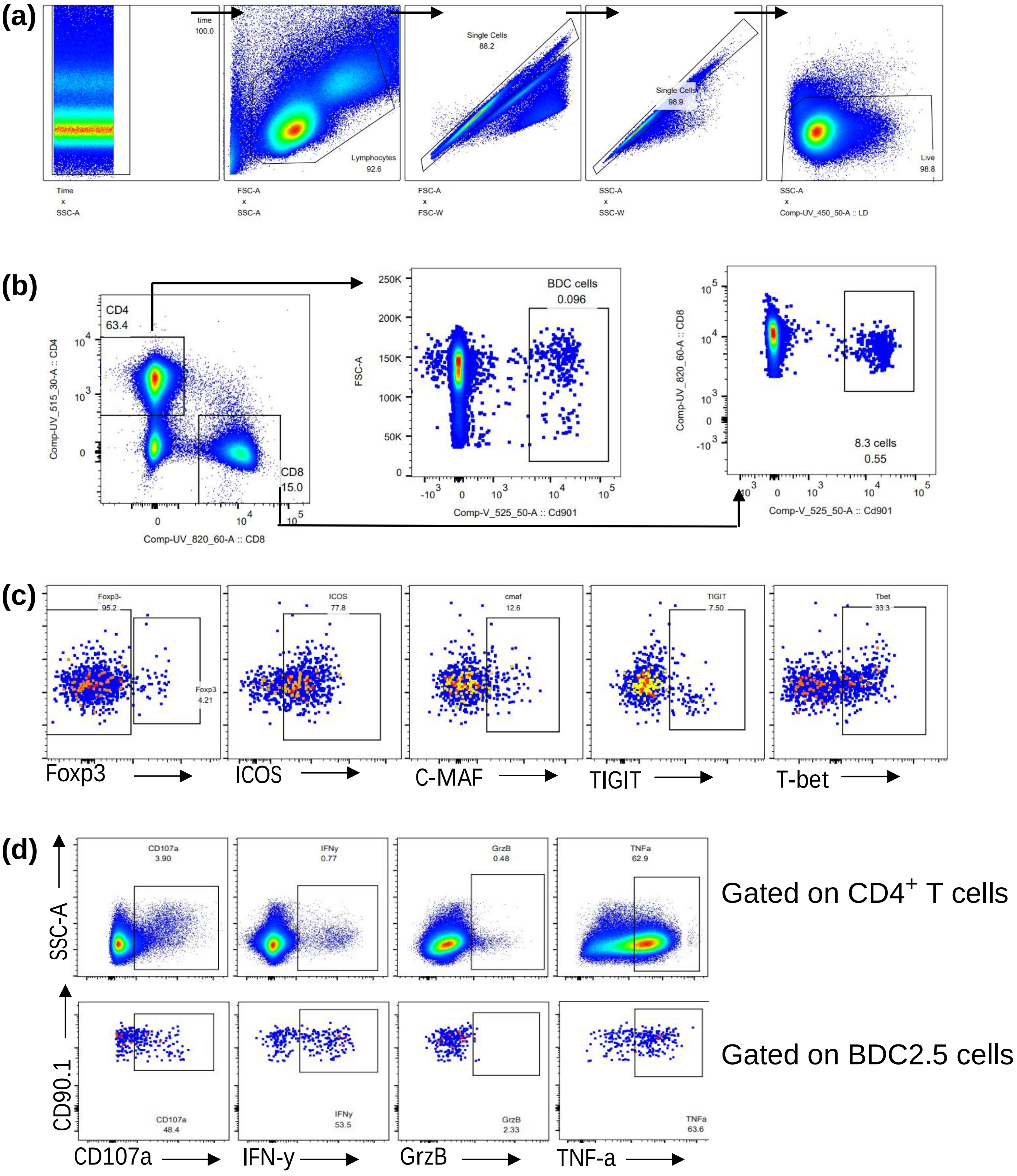

Supplementary figure 8. Flow cytometry gating strategy. (a) Exclusion of debris, doublets and dead cells. **(b)** Identification of transferred BDC2.5 and 8.3 cells. **(c)** Exmaple gating of transcription factors and surface markers at day 4 in BDC2.5 cells. **(d)** example gating of cytokines and secretory ganules after *ex vivo* stimulation with PMA/Ionomycin in bulk $CD4^+$ T cells and BDC2.5 cells.
